# Plin2 Inhibits Cellular Glucose Uptake through Interactions with SNAP23, a SNARE Complex Protein

**DOI:** 10.1371/journal.pone.0073696

**Published:** 2013-09-06

**Authors:** Subramanian Senthivinayagam, Avery L. McIntosh, Kenneth C. Moon, Barbara P. Atshaves

**Affiliations:** 1 Biochemistry and Molecular Biology, Michigan State University, East Lansing, Michigan, United States of America; 2 Veterinary Physiology and Pharmacology, Texas A&M University, College Station, Texas, United States of America; Fundação Oswaldo Cruz, Brazil

## Abstract

Although a link between excess lipid storage and aberrant glucose metabolism has been recognized for many years, little is known what role lipid storage droplets and associated proteins such as Plin2 play in managing cellular glucose levels. To address this issue, the influence of Plin2 on glucose uptake was examined using 2-NBD-Glucose and [^3^H]-2-deoxyglucose to show that insulin-mediated glucose uptake was decreased 1.7- and 1.8-fold, respectively in L cell fibroblasts overexpressing Plin2. Conversely, suppression of Plin2 levels by RNAi-mediated knockdown increased 2-NBD-Glucose uptake several fold in transfected L cells and differentiated 3T3-L1 cells. The effect of Plin2 expression on proteins involved in glucose uptake and transport was also examined. Expression of the SNARE protein SNAP23 was increased 1.6-fold while levels of syntaxin-5 were decreased 1.7-fold in Plin2 overexpression cells with no significant changes observed in lipid droplet associated proteins Plin1 or FSP27 or with the insulin receptor, GLUT1, or VAMP4. FRET experiments revealed a close proximity of Plin2 to SNAP23 on lipid droplets to within an intramolecular distance of 51 Å. The extent of targeting of SNAP23 to lipid droplets was determined by co-localization and co-immunoprecipitation experiments to show increased partitioning of SNAP23 to lipid droplets when Plin2 was overexpressed. Taken together, these results suggest that Plin2 inhibits glucose uptake by interacting with, and regulating cellular targeting of SNAP23 to lipid droplets. In summary, the current study for the first time provides direct evidence for the role of Plin2 in mediating cellular glucose uptake.

## Introduction

When chronic over-nutrition leading to obesity occurs, excess lipids that are released from adipose tissue are stored as ectopic fat in the liver and skeletal muscle (also heart and pancreas). Increased levels of lipid metabolites such as diacylglycerols and ceramides impair insulin signaling, resulting in reduced cellular glucose uptake and insulin sensitivity [1]. To protect cells from the lipotoxic effects and cellular dysfunction of lipid metabolites, intracellular lipids are stored in lipid droplets, vesicles composed of a neutral lipid core surrounded by a phospholipid monolayer with embedded proteins coating the surface. Lipid droplets are highly dynamic organelles involved in various cellular functions [2]. Understanding cell-specific regulation of lipids reserved in lipid droplets is an active area of research and cell type often defines the proteins associated with lipid droplets. The perilipin (Plin) family of proteins are lipid droplet-associated proteins related through sequence homology and affinity for lipid droplets [3–5]. Plin1 (formerly known as perilipin) and Plin4 (S3-12) are found primarily in adipocytes and steroidogenic cells. Plin2 and Plin3 (previously TIP47, M6PRBP) are both ubiquitously expressed in all cell types with high levels of Plin2 observed in hepatocytes and skeletal muscle cells. Plin5 (also known as OXPAT, PAT1, LSDP5), is found primarily in cells with high energy requirements including myocytes and hepatocytes. Plin1 and Plin2 are constitutively located on the lipid droplet surface while Plin 3-5 can be found in both cytosolic and lipid droplet compartments [6–9]. Plin1 is the most studied lipid droplet protein with known function involving TG hydrolysis [3–5], but the full physiological significance of the rest of the Plin family remains less defined. With regard to Plin2, several reports describe increased TAG accumulation and lipid droplet formation when Plin2 is overexpressed in cells [10–13]. Conversely, knockdown of Plin2 in macrophages was shown to decrease cellular lipids and lipid droplet size and number [10]. In studies with Plin1 knockout mice, Plin2 was up-regulated and replaced Plin1 on the surface of lipid droplets without replacing Plin1’s hydrolytic function [14], yet in other work, Plin2 regulated access of the lipase ATGL (adipose triglyceride lipase) to the lipid droplet surface to influence TAG hydrolysis [12]. These results, along with the fact that Plin2 binds lipids such as cholesterol [15–17], fatty acids [15,18], and phospholipids [19] with high affinity, suggest that Plin2 may play an important role in maintaining lipid homeostasis. In keeping with this, studies with several mouse models revealed that Plin2 ablation yields mice with reduced hepatic lipids that are resistant to diet-induced fatty liver and adipose inflammation [20,21] without changes in TAG synthesis or fatty acid uptake, synthesis, or β-oxidation. Indirect evidence that Plin2 is also involved with managing glucose levels comes from several studies including work with the Zucker diabetic rat model which showed increased levels of Plin2 in skeletal muscle correlated with insulin resistance when fed a high fat diet [22]. Moreover, diabetic patients with increased Plin2 expression exhibited reduced insulin-stimulated glucose uptake [22]. In other work, mice treated with anti-sense Plin2 oligonucleotides were protected against diet-induced insulin resistance when fed high fat diets [23]. Chang et al. demonstrated that mice deficient in Plin2 and crossed with ob*/*ob mice showed an improvement in insulin resistance in the liver and skeletal muscle [24]. However, the presence of an active Plin2 truncated protein (designated Δ2,3-ADPH) in the Plin2 knockout model confounded interpretation of this work [25]. The above studies indicated a connection between altered Plin2 expression and the development of insulin resistance, yet direct evidence of Plin2 involvement was not provided. The present work was undertaken to address this issue by examining the influence of Plin2 on SNARE-mediated glucose transport and metabolism.

Of central importance in determining cellular insulin sensitivity is the ability of cells to take up and utilize glucose. Cellular glucose uptake is mediated through the action of two major types of glucose transporters namely: facilitative glucose transporters (GLUT) and sodium-glucose transporters (SGLT) [26]. Several isoforms of GLUT exist including the ubiquitously expressed GLUT1 and also GLUT4, predominantly expressed in muscles and adipocytes [27] and regulated by insulin [28,29]. Under basal conditions more than 90% of GLUT4 resides in the cytoplasm within glucose transporter vesicles (GSV). Upon insulin stimulation these vesicles translocate to, then dock and fuse with the plasma membrane to enhance GLUT4 availability and facilitate glucose entry into the cell [30]. These events are mediated by various SNARE proteins that have also been shown to target to lipid droplets to regulate lipid droplet fusion and growth [31,32], suggesting that partitioning of SNARE proteins between lipid droplets and the plasma membrane represents a potential point of control in the regulation of both glucose uptake and lipid droplet biogenesis. Since Plin2 is known to promote lipid droplet formation and growth [20,33,34], the role of Plin2 in SNARE-mediated glucose uptake and transport was examined using laser scanning confocal microscopy, radioligand assays, co-immunoprecipitation, and FRET techniques. The data presented herein provides the first direct evidence of the influence of Plin2 on glucose uptake and SNARE protein dynamics, in keeping with a key role for Plin2 in glucose transport and metabolism.

## Materials and Methods

### Materials

DMEM, 2-NBD-glucose, and Opti-MEM were purchased from Life Technologies-Invitrogen (Carlsbad, CA). The glucose transport inhibitor cytochalsin B was purchased from Sigma (St. Louis, MO). [^3^H]-2-deoxyglucose was purchased from American Radiochemicals Inc., (Saint Louis, MO). Commercially available polyclonal antibodies made in rabbit were purchased from the following vendors: anti-Syntaxin-5 and mouse monoclonal anti-VAMP-4 were from Santa-Cruz biotechnologies (Santa-Cruz, CA); anti-SNAP23 was from Abcam (Cambridge, MA); anti-insulin receptor was from Abbiotech, LLC (San Diego, CA); anti-Plin1 was purchased from Thermo Scientific-Affinity Bioreagents (Rockland, IL), and anti-FSP27 was from Lifespan Biosciences (Seattle, WA). Anti-rabbit polyclonal anti-serum to Plin2 was developed in house as described earlier [35]. Anti-GLUT1 used for immunoprecipitation experiments and immunoblotting was from Fabgennix (Frisco, TX) and Epitomics (Burlingame, CA), respectively. Goat anti-SNAP23 polyclonal used in immunofluorescence studies was purchased from Everest Biotech (Ramona CA). The Cy3-labeled anti-goat secondary antibody used for FRET studies was purchased from Jackson Immuno Research Laboratories, Inc. (West Grove, PA) while the Cy5-labeled anti-rabbit secondary antibody was from Life Technologies-Invitrogen (Carlsbad, CA). All reagents and solvents used were of the highest grade available and were cell culture tested.

### Cell Culture

L cell fibroblasts were derived from NCTC clone 929 (L cell, derivative of Strain L, ATCC® CCL-1™). Cells were maintained in high glucose DMEM containing 10% FBS and antibiotics (100 units/ml penicillin and 100 units/ml streptomycin) under 5% CO_2_ at 37^o^C. Plin2 overexpressing L cells were generated by stable transfection using a plasmid containing the full coding region of mouse Plin2 cDNA N-terminally fused to CFP as described elsewhere [13]. It should be noted that the N-terminal CFP tag had no effect on Plin2 targeting to lipid droplets as assessed by laser scanning confocal microscopy, consistent with earlier reports [34]. Mock-transfected control cells (designated as controls) were created by stable transfection into L cells using the empty CFP vector. For glucose uptake studies, control cells were seeded in 2-well chamber slides (Nunc, Naperville, IL) at a density of 5x10^5^ cells/well. Undifferentiated 3T3-L1 pre-adipocytes (ATCC® CL-173™) were maintained in DMEM containing 10% FBS and antibiotics (100 units/ml penicillin and 100 units/ml streptomycin) under 5% CO_2_ at 37^o^C. For glucose uptake studies and protein analysis with 3T3-L1 cells, preadipocytes were differentiated into adipocytes following standard procedures. In brief, confluent 3T3-L1 cells were maintained in the presence of serum containing DMEM supplemented with dexamethasone (1 µM), methylisobutylxanthine (500 µM) and insulin (1 µg/ml) for 48 hrs and then switched to DMEM containing insulin (1 µg/ml), replacing media every 48 hrs. After 6 days, the majority of cells were differentiated into adipocytes.

### Lipid analysis

Neutral lipids were extracted from CFP-ADRP and control cell homogenates and resolved into individual lipid classes as described earlier [36,37]. In brief, samples extracted with n-hexane: 2-propanol 3:2 (v/v) were resolved by lipid groups into cholesterol (Chol), free fatty acids (FFA), triacylglycerol (TG), cholesteryl esters (CE), and total phospholipids (PL) using Silica gel G TLC plates developed in petroleum ether-diethyl ether-methanol-acetic acid (90:7:2:0.5, v/v/v/v). For resolution of diacylglycerols (DG), the following solvent system was used: petroleum ether-diethyl ether-glacial acetic acid (280:120:4 v/v/v/v). All lipids were identified by comparison to known standards. Levels of neutral lipids, (DG, TG, CE) were analyzed by the method of Marzo et al. [38]. Protein concentration was determined by the method of Bradford from the dried protein extract residue digested overnight in 0.2 M KOH [39]. Lipids were stored under an atmosphere of N_2_ to limit oxidation and all glassware was washed with sulfuric acid-chromate before use.

### 2-NBD-Glucose uptake

Glucose uptake studies were performed on cells (transfected L cell fibroblasts or differentiated 3T3-L1 cells) with altered Plin2 levels using the fluorescent glucose analogue, 2-NBD-Glucose, at room temperature using a modification of methods described elsewhere [40,41]. In brief, cells grown in 2-well chamber slides were washed twice with phosphate-buffered saline and placed on an Olympus FluoView 1000 Laser Scanning Confocal Microscope (Olympus, America, Inc., Center Valley, CA) equipped with an IX81 automated inverted microscope and operated with Fluoview software. To acquire images, fluorescent probes were excited using the FluoView’s 488nm Argon ion laser for NBD label and the 458 nm Argon line for CFP. Fluorescence emission of 2-NBD-Glucose was collected using a 505-525 nm band pass filter. During image acquisition, cells were exposed to the light source for minimal time periods to minimize photobleaching effects. A field of 20-25 cells was selected for analysis and 2-NBD-Glucose (50 µM) was added to start the uptake process. During the time course, real time fluorescent images were acquired every 30 seconds until saturation was reached. Uptake into the cell was measured as an increase in 2-NBD-Glucose fluorescence intensity inside the cell using Metamorph image analysis software (Molecular Device, Sunnyvale, CA). Exclusive thresholding of levels were adjusted to remove the extracellular fluorescence. Intensity values were plotted using Sigmaplot 11.0 (Systat Software, Inc., San Jose, CA) and curve-fitted by non-linear regression analysis using the following formula f = a (1-e^-bt^) where “a” represented the Fmax, corresponding to the maximal capacity of glucose uptake; “t” was time, and “b” was the apparent rate constant, representing the rate of approach to Fmax. Initial rates were calculated from the linear portion of the 2-NBD-Glucose uptake curve (0-5 min) fitted by linear regression analysis.

### Radioactive 2-deoxyglucose uptake

Glucose uptake was studied using [^3^H]-2-deoxyglucose following the method of Nedachi et al. [42]. Briefly, L cell control and Plin2 overexpression cells were plated in 6 well dishes (10^5^ cells/well) and serum starved overnight. Cells were washed twice with Krebs-Ringer-HEPES (KRH) buffer and uptake was initiated by the addition of radioactive stock solution (19.5 mM cold 2-deoxyglucose with 5 µCi/ml [^3^H[-2-deoxyglucose) to give a final concentration of 6.5 mM 2-deoxyglucose (0.5 µCi [^3^H[-2-deoxyglucose/well) in each well. After 5 minutes, the reaction was stopped by addition of ice cold KRH buffer. Cells were washed three times with ice-cold KRH buffer, then lysed with lysis buffer (10 mM Tris pH 7.4, 150 mM NaCl, 5 mM EDTA, 1.0% triton X-100, 0.4% SDS) and collected to measure radioactivity per well.

### Cytochalasin B, glucose, and insulin effects on 2-NBD-Glucose uptake in L cell fibroblasts

Glucose uptake was performed in the presence of either cytochalasin B, a well-known glucose transport inhibitor [43], unlabeled D-glucose, or insulin to examine the mechanism of glucose uptake in L cells. To determine if glucose uptake in L cells was GLUT-mediated, 2-NBD-Glucose (50 µM) was added to cells pre-treated with and without cytochalasin B (100 µM) for 30 minutes and fluorescence intensity was measured at time 0 and 30 min after addition of 2-NBD-Glucose. Relative integrated intensities were calculated as described in the previous section. Competitive inhibition of 2-NBD-glucose uptake by excess unlabeled D-glucose was examined by incubating cells with 2-NBD-glucose and D-glucose (25mM) for 30 minutes. Next, to determine if glucose uptake in L cells was insulin-induced, 2-NBD-Glucose uptake in cells pre-treated with insulin (100 nM) for 30 minutes followed by addition of 2-NBD-Glucose (50 µM). Fluorescent images were acquired for 30 minutes. Increases in fluorescence intensity, Fmax and initial rate were calculated as described above.

### Western blot analysis

Expression levels of proteins involved in glucose transport (insulin receptor, GLUT1), lipid droplet function (Plin2, Plin1, FSP27), and vesicular transport (VAMP4, SNAP23, syntaxin-5) were assessed by Western blot analysis and normalized to the mean expression of GAPDH as described in [44]. For each blot, equal amounts of cell homogenate (15-20 µg protein, depending on the cellular expression level of the protein of interest) was loaded onto tricine gels (10%) and run on a Mini-Protean II cell (Bio-Rad lab, Hercules, CA) system at constant amperage (30 mA per gel) for approximately 1.5 to 2 hrs. Proteins were then transferred electrophoretically to nitrocellulose membranes (Bio-Rad) at constant voltage (90 V) for 1.5 hrs. After transfer, the blots were stained with Ponceau to confirm protein transfer and constant protein loading [45,46]. Blots were then processed using the Western Breeze Chemiluminescent Immunodetection kit (Life technologies-Invitrogen, Carlsbad, CA) to detect relative levels of the protein of interest and a housekeeping gene (GAPDH or actin) following the manufacturer’s instructions. Scanned images of the Western blots (8-bit gray scale density) were used for densitometric analysis using NIH Scion Image to obtain relative protein levels expressed as integrated density values. The integrated density, representing the mean density of pixels multiplied by the area was determined from equal sized rectangles drawn around the bands of interest minus the background to remove non-specific antibody staining.

### RNA Interference (siRNA)

Plin2 siRNA (sense-5′-AACGUCUGUCUGGACCGAAUA-3′ and the corresponding antisense) sequences described in [47] were synthesized from Dharmacon (Lafayette, CO). A non-targeting control siRNA was purchased from Dharmacon. In L cells, the siRNA transfection was performed using opti-MEM and lipofectAMINE 2000 as per the manufacturer’s instructions. Briefly, cells (0.4 x 10^6^ cells/ well) in 6-well plates (Nunc, Naperville, IL) were transfected with either the non-targeting control siRNA (50 nM) or the Plin2 siRNA (50 nM). Untransfected control cells plated at the same density were maintained simultaneously. Six hours after transfection, the media was changed to complete growth medium. With differentiated 3T3-L1 cells, the siRNA transfection was performed with control or Plin2 siRNA (20 nM) using DeliverX plus siRNA reagent (Affymetrix, Santa Clara, CA) following the manufacturer’s protocol. One well for each treatment in both L cells and 3T3-L1 cells was trypsinized and seeded into 2-well chamber slides and 6-well plates for uptake studies and Western blotting, respectively at 48 hrs (3T3-L1 cells) or 72 hours (L cells) post transfection.

### Colocalization and fluorescence resonance energy transfer (FRET) imaging

The intracellular localization and partitioning of SNAP23 with Plin2 and GLUT1 was examined by colocalization and FRET imaging studies following procedures described earlier [13,15]. Briefly, the colocalization studies were performed with Plin2 overexpression and control cells seeded at a density of 50,000 cells/well in 4-well chamber slides (EZ Slide, Millipore, Billerica, MA) incubated overnight at 37°C. The cells were fixed using cold acetone/ethanol (70: 30 v/v) and washed with PBS. The cells were blocked with 2% BSA and then incubated with primary antibodies for 1 hr at room temperature with either goat anti-SNAP23 (1:25) and rabbit anti-Plin2 (1:50) or goat anti-SNAP23 (1:25) and rabbit GLUT1 (1:25). After extensive washing with PBST (0.05% Tween100 in PBS), a mixture of secondary reagents consisting of Cy3 or Cy5 labeled secondary antibodies (at 1:100 dilutions) in PBS was added to each set and incubated for 1 hr at room temperature. Cells were then washed with PBS and mounted with coverslips using fluorogel mounting medium (Electron Microscopy Science, Hatfield, PA). Cells were also stained with Cy3 and Cy5 alone in the absence of primary antibodies as a standard control. Cell images were sequentially acquired on an Olympus FluoView 1000 Laser Scanning Confocal Microscope using 559 nm excitation, 575/50 filter (green channel) to view the Cy3 emission and 635 nm excitation, 725/30 filter (red channel) for the Cy5 emission. Co-localization of the Cy3- and Cy-5 signals was obtained using the Olympus Fluoview software where the confocal images from the green and red channels were merged and appeared yellow where superimposition occurred (red and green are additive and yield yellow to orange in RGB color space). The percentage of green and red co-localization in cells was calculated based on the following equations:

Cred=∑iRi,coloc∑iRi

Cgreen=∑iGi,coloc∑iGi

where Σ R_*i,coloc*_ is the sum of intensities of all red pixels which also have a green component; ΣR_*i*_ is the sum of intensities of all red pixels in the image; ΣG_*i,coloc*_ is the sum of intensities of all green pixels which also have a red component; and ΣG_*i*_ is sum of intensities of all the green pixels in the image.

For the FRET imaging studies, cells were processed as for the co-localization studies to measure the increase in donor (Cy3) emission upon photobleaching of the acceptor (Cy5) as described in [13]. In brief, the FRET experiment was performed by measuring the fluorescence emission of the Cy3 donor through the 575/50 nm filter upon excitation at 559 nm before and after photobleaching of the Cy5-acceptor at 633 nm. Before performing the FRET experiment multiple controls were used to avoid interference from non-specific fluorescence: (i) Non-labeled cells were used to set the gain and black level in the Cy3 channel so that no cellular autofluorescence was detected in the donor Cy3 channel while maintaining maximum dynamic range; (ii) Cells labeled with Cy5 were excited at 559 nm to show no observed fluorescence in the donor Cy3 channel by adjusting the gain and black level in Cy5 channel to suppress Cy3 fluorescence bleed-through; and (iii) Donor cells (Cy3-labeled) without Cy5 present were subjected to bleaching to check for non-specific fluorescent increases in the donor channel and also to optimize bleaching parameters so that the donor intensity was not affected by bleaching. Additionally, one or two cells during the FRET experiment were left unbleached to serve as in-field bleaching control. To calculate the FRET efficiency (*E*), representing the efficiency of energy transfer between donor and acceptor, the following equation was used: *E* = 1-(*I*_DA_/*I*_D_) where *I*_DA_ is donor fluorescence intensity before acceptor photobleaching and *I*_D_ is the donor fluorescence intensity after acceptor photobleaching. An average *E* value was calculated from Cy3 fluorescence emission increase after photobleaching. The intermolecular distance *R* between SNAP23 and Plin2 or SNAP23 and GLUT1 was calculated from the equation *E* = 1/(1-(*R*/*R*_o_)^6^), where *E* is experimentally determined and *R*_o_ is the Foster radius for the Cy3-Cy5 FRET pair, previously determined as 51 Å. For the FRET efficiency images, analysis was performed in MetaMorph 7.5 (Molecular Devices, Sunnyvale, CA) as described in with images filtered to remove randomized noise by using a low pass filter using a 4 x 4 pixel setting. The filtered images of the donor emission before acceptor photobleaching were subtracted from the image after acceptor photobleaching. The resultant image was divided by the image of donor emission after acceptor photobleaching and multiplied by 100 to show the grayscale FRET efficiencies. A FRET overlay was created from this image and pseudo-colored in order to visualize regions of higher and lower FRET where black indicated little to no efficiency and red to yellow represented efficiencies greater than 80%.

### Co-immunoprecipitation studies

The Catch and Release v2.0 system from Millipore (Billerica, MA) was used for co-immunoprecipitation (co-IP) studies following the manufacturers’ protocol. Briefly, cell lysates from Plin2 overexpressing or control cells were incubated with the spin column resin, specific antibody (1-4 µg), and the antibody affinity capture ligand provided in the kit (10 µl) overnight at 4^0^C with shaking. The following day, unbound fractions were separated by centrifugation, followed by washing and the bound complex was eluted. Eluate proteins were analyzed by Western blotting with n=3 blots for each co-IP. A negative control (rabbit IgG) was used to assess nonspecific binding.

### Statistical Analysis

All values were expressed as the means ± S.E. Student’s *t* tests were performed when comparisons are made between two groups using Graphpad Prism (San Diego, CA). When more than two groups were compared one-way analysis of variance (ANOVA) followed by multiple comparison test using the Holm-Sidak method. Values with *p* < 0.05 were considered statistically significant.

## Results

### Dose dependence of 2-NBD-Glucose uptake in mouse L cell fibroblasts

**Figure 1:**
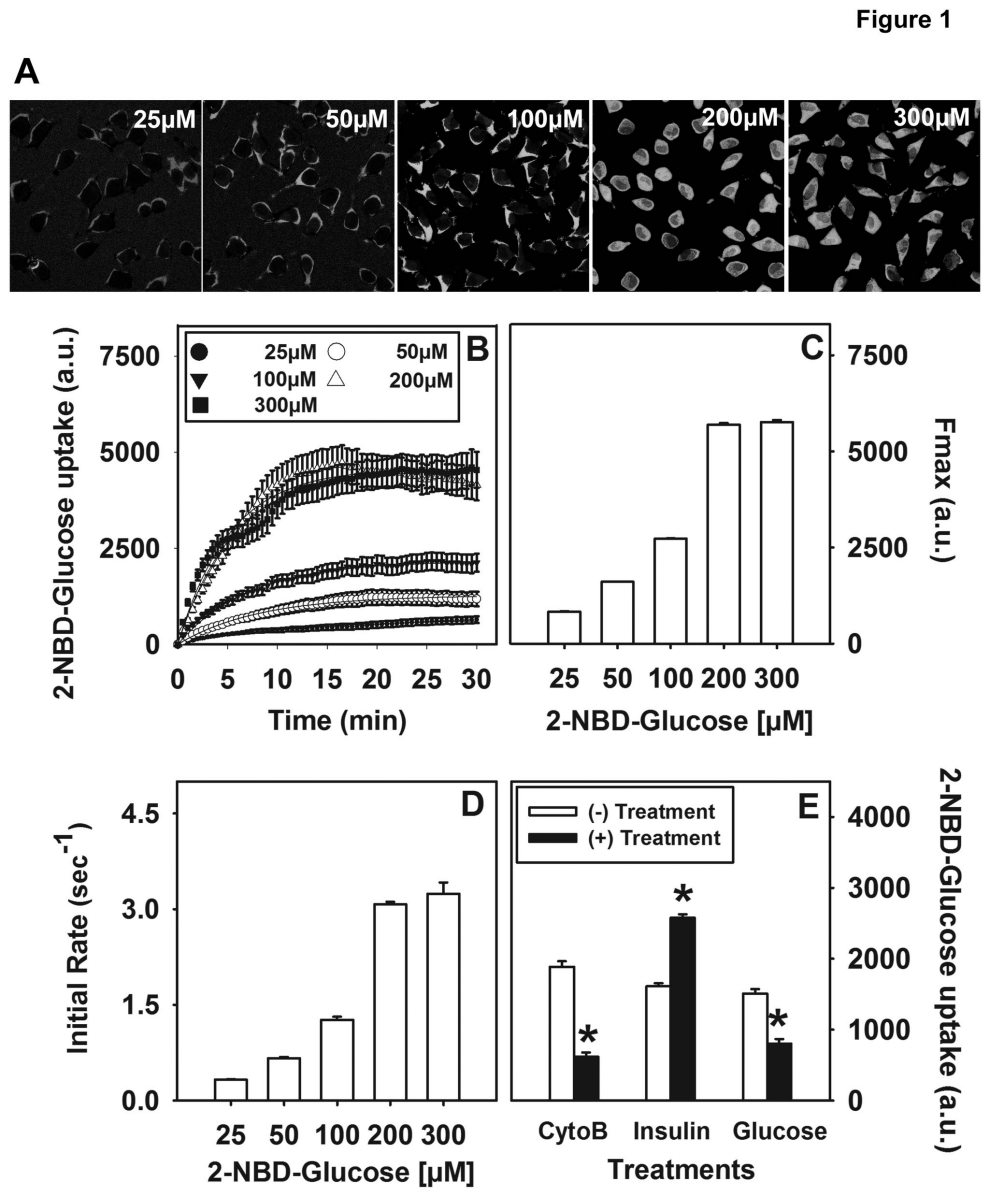
Dose response and effect of cytochalsin B and insulin on 2-NBD-Glucose uptake into L cells. Representative confocal images of L cells labeled with different doses of 2-NBD-Glucose (50-300 µM) are shown (A). Uptake of 2-NBD-Glucose (B) and the time-course parameters Fmax (C) and initial rate (D) were calculated as described in the Methods section. The effect of the glucose transport protein inhibitor, cytochalsin B, insulin, and unlabeled glucose on 2-NBD-Glucose uptake after 30 min incubation was determined (E). Increases in fluorescence intensity were calculated and expressed as arbitrary units (a.u.). Values represent mean ± SEM. (*) indicates p < 0.05 as compared to control.

The dose dependence of 2-NBD-glucose uptake was performed using laser scanning confocal microscopy (LSCM) on L cell fibroblasts. Despite their fibroblast-like morphology, L cells provide a useful model to study glucose uptake and transport since they were derived from mouse subcutaneous adipose and areolar tissue (ATCC number CCL-1) and contain all the necessary machinery for glucose utilization [48] and cellular metabolism [49–54] to mimic similar cell types commonly used to study glucose metabolism. Additionally, the large data set of information available and the fact that the abundance of Plin2 in L cells could easily accommodate increased or decreased expression without issues of cell toxicity or lack of detection made L cells the model of choice. Representative images of L cells labeled with 2-NBD-Glucose for 20 min at each concentration (25-300 µM) is shown in Figure 1A. 2-NBD-Glucose uptake at increasing concentrations of the probe was assessed over 30 min to determine the optimal concentration of 2-NBD-glucose before saturation (Figure 1B). Fmax, corresponding to the maximal capacity of glucose uptake (Figure 1C) and the initial rate of uptake (Figure 1D) was observed to increase up to 2-fold when L cells were incubated with 25 µM to 200 µM 2-NBD-glucose. At levels above 200 µM no further increase was observed in either Fmax or initial rates, indicating that the process had reached saturation. As a result, a concentration mid-phase in the linear increase (50 µM) was chosen for subsequent glucose uptake studies.

### Effect of the GLUT inhibitor cytochalasin B and insulin on 2-NBD-Glucose uptake in L cell fibroblasts

Since glucose uptake is mediated through the action of glucose transporters [26], it was important to verify that the fluorescent glucose analogue 2-NBD-Glucose was GLUT-mediated in our L cell model. Therefore, 2-NBD-Glucose uptake studies were performed in the presence or absence of the glucose transporter inhibitor, cytochalasin B [43] and the extent of uptake was calculated as described in the Methods section. Analysis of multiple experiments revealed that 2-NBD-Glucose uptake was significantly reduced 65% (p < 0.001) in the presence of cytochalasin B, in keeping with a GLUT-mediated mechanism (Figure 1E). Moreover, in the presence of D-glucose 2-NBD-Glucose uptake was significantly reduced (Figure 1E), suggesting that the excess unlabeled glucose was able to competitively inhibit 2-NBD-glucose. These results further confirmed 2-NBD-glucose uptake was mediated through the physiological glucose transport system. The effect of insulin, a known physiological stimulator of glucose uptake, on 2-NBD-Glusose uptake was next examined. Since L cells contain both high and low affinity insulin binding sites [54] it was not surprising to see that insulin treatment induced a 1.6-fold increase in 2-NBD-Glucose uptake (Figure 1E, p < 0.05). Taken together, these results indicated that 2-NBD-Glucose uptake in L cells was insulin responsive and GLUT-mediated.

### Effect of altered Plin2 expression on 2-NBD-Glucose uptake in transfected L cells

**Figure 2:**
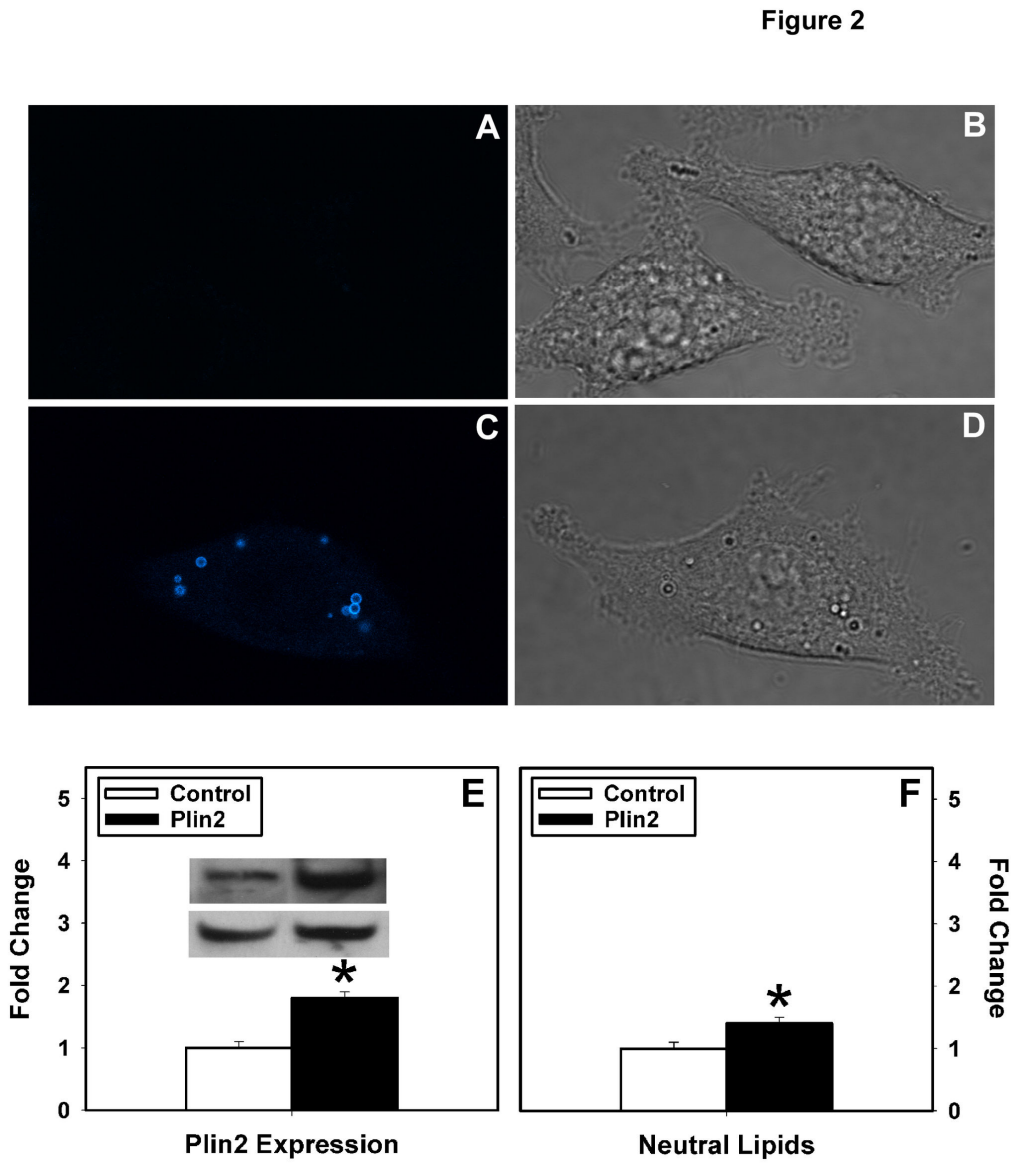
Effect of Plin2 overexpression in transfected Lcells. L cells were transfected with empty vector to show little to no fluorescence in lipid droplets (A) that were evident by light microscopy (B). Lipid droplet targeting of the CFP-Plin2 construct was confirmed by confocal microscopy showing CFP-labeled lipid droplets in Plin2 overexpressing cells (C) that were also visible by light microscopy (D). Relative expression of Plin2 (E) and neutral lipid content (F) in control (open bar) and Plin2 overexpression (closed bar) cells were measured as described in Methods. (*) indicates p<0.05 as compared to control. Insets: Representative Western blots showing relative protein expression of Plin2 and GAPDH in control and Plin2 overexpression cells.

**Figure 3:**
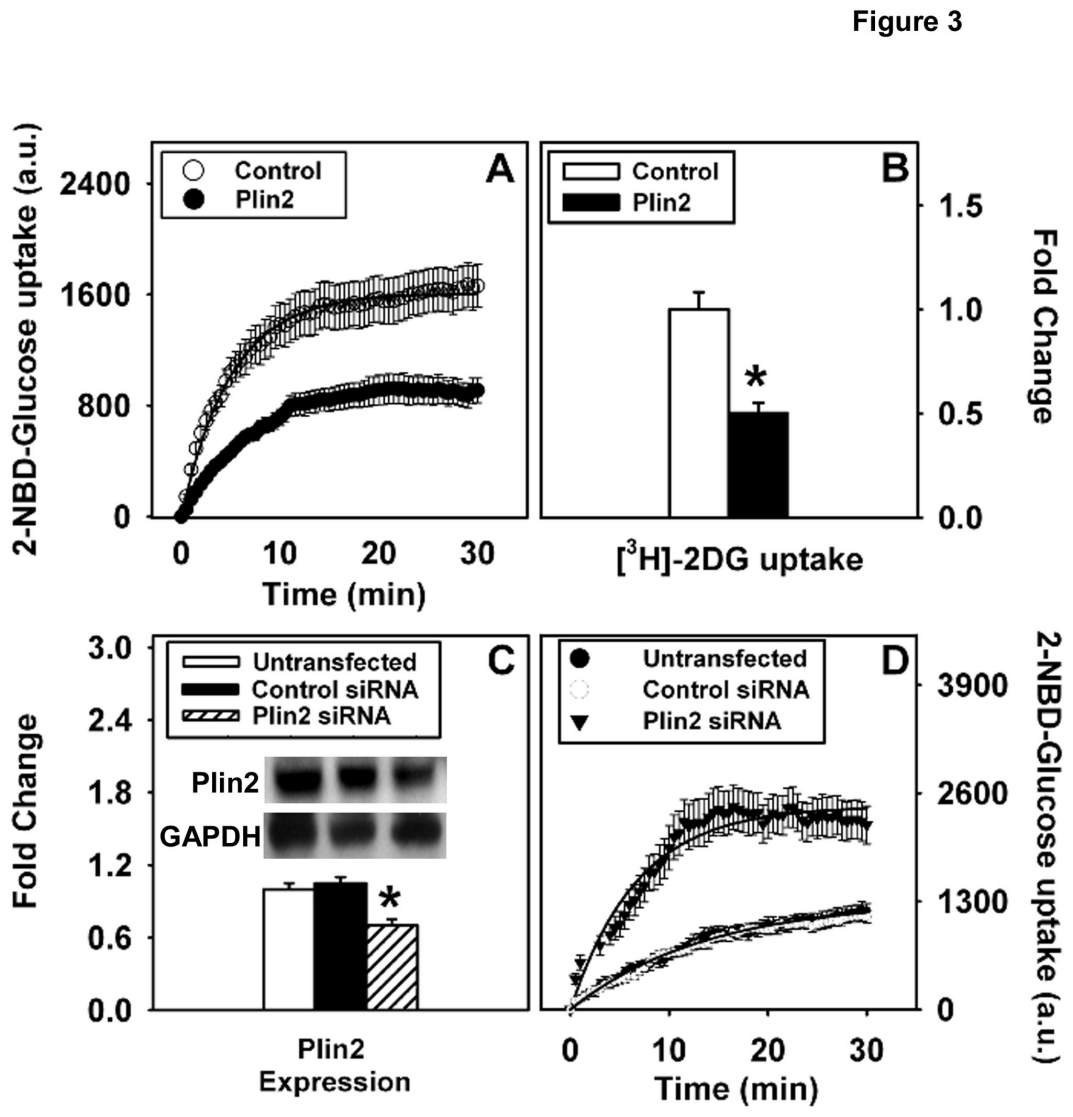
Effect of altered Plin2 expression on 2-NBD-Glucose uptake in L cells. 2-NBD-Glucose uptake (A) in Plin2 overexpression (closed circles) and control (open circle) cells was measured as an increase in fluorescence intensity expressed in arbitrary units (a.u.) as described in the Methods section. [^3^H]-2-deoxyglucose uptake (B) was measured in control (open bar) and Plin2 overexpression (closed bar) cells as a fold-change in radioactivity relative to control cells. Relative expression levels of Plin2 after siRNA-mediated Plin2 knock down (C) and 2-NBD-Glucose uptake (D) in cells that were untransfected (closed circles), treated with control siRNA (open circle), or Plin2 siRNA (closed square) was also measured, normalized to the housekeeper gene GAPDH. Values represent mean ± SEM (n=3). (*) indicates p < 0.05 as compared to control. Insets: Representative Western blots showing relative protein expression of Plin2 and GAPDH.

To examine the effect of Plin2 on cellular glucose uptake, 2-NBD-Glucose uptake was performed in cells with altered Plin2 expression. Plin2 overexpression cells were developed by stably transfecting L cells with mouse cDNA encoding for CFP-labeled Plin2 as described earlier [13]. Lipid droplets in control cells transfected with the empty vector were visible by light microscopy (Figure 2B) but exhibited little to no fluorescence above background (Figure 2A). In contrast, transfection of L cells with CFP-Plin2 construct resulted in fluorescently labeled lipid droplets (Figure 2C) that were also able visible by light microscopy (Figure 2D). These results demonstrated that the N-tagged CFP-Plin2 did not affect lipid droplet targeting, consistent with previous reports in 3T3-L1 cells with GFP-labeled Plin2 [34]. Levels of Plin2 in the transfected clones were 1.8-fold higher than mock-transfected L cells as shown by Western blotting using antibodies against Plin2 (Figure 2E, p<0.01). A similar fold increase (1.6-fold) was observed using antibodies against CFP to detect the CFP-labeled Plin2 (data not shown). In addition, levels of neutral lipids (diacylglycerols, triacylglycerols, cholesteryl esters) were significantly increased 1.4-fold in the Plin2 overexpression cells (Figure 2F, p<0.05), consistent with previous reports in other cell types [11,13,34]. When Plin2 overexpressing cells were incubated with 2-NBD-Glucose, analysis of multiple uptake experiments showed a 70% reduction in 2-NBD-Glucose uptake (p < 0.001) as compared to mock-transfected control L cells (Figure 3A). These results reflected a 42% decrease in Fmax and a 53% decrease in the initial rate of cellular 2-NBD-Glucose uptake. Parallel studies with [^3^H]-2-deoxyglucose confirmed these results (Figure 3B). To verify the Plin2 inhibitory affect on glucose uptake, RNAi-mediated knock down of Plin2 in L cells (Figure 3C) and in differentiated 3T3-L1 cells (Table 1) was performed followed by glucose uptake experiments (Figure 3D). A significant 35% reduction in Plin2 expression in L cells transfected with Plin2 siRNA (Figure 3C, P < 0.05, n=3) resulted in a 1.9-fold increase in 2-NBD-Glucose uptake after 30 min (Figure 3D, Table 1, p<0.05). Initial rates were similarly increased in the knock down cells (Table 1, p < 0.05). Control siRNA cells did not show increased glucose uptake but were comparable to those performed with untransfected control cells (Table 1), indicating that the associated increase in uptake observed with Plin2 knock down cells was Plin2-specific. To verify that results with L cells were not cell specific, Plin2 was knocked down in differentiated 3T3-L1 cells, an established model of adipocytes and 2-NBD-Glucose uptake experiments were repeated. Similar to L cells, Plin2 knock down in differentiated 3T3-L1 cells resulted in a 3-fold increase in 2-NDB-Glucose maximal uptake and initial rate of uptake (Table 1, p < 0.05). In summary, studies with Plin2 overexpression cells confirmed that increased Plin2 expression inhibited GLUT-mediated 2-NBD-Glucose uptake into L cells. Conversely, RNAi-mediated reduction of Plin2 significantly increased cellular 2-NBD-Glucose uptake in both L cells and 3T3-L1 differentiated cells. Taken together, these results indicated a negative correlation between Plin2 expression and cellular glucose uptake.

**Table 1:** Effect of Plin2 knock down on 2-NBD-Glucose uptake in L cells and differentiated 3T3-L1 cells.

Cells	L-cells		3T3-L1 cells	
	Fmax(a.u.)	Initial Rate (sec^-1^)	Fmax(a.u.)	Initial Rate (sec^-1^)
Untransfected control	1257 ± 18	0.23 ± 0.01	918 ± 19	0.15 ± 0.01
Control siRNA	1313 ± 20	0.21 ± 0.01	841 ± 7	0.10 ± 0.08
Plin2 siRNA	2433 ± 38*	0.52 ± 0.01*	3118 ±187*	0.5 ± 0.1*

The effects of Plin2 knock down on 2-NBD-glucose uptake parameters, Fmax and initial rate, were determined as described in Method section. Increases in fluorescence intensity were calculated and expressed as arbitrary units (a.u.). Values represent mean ± SEM. (*) indicates p < 0.05 as compared to untransfected control cells.

### Effect of Plin2 overexpression on key proteins involved in lipid droplet formation and glucose uptake and transport

**Figure 4:**
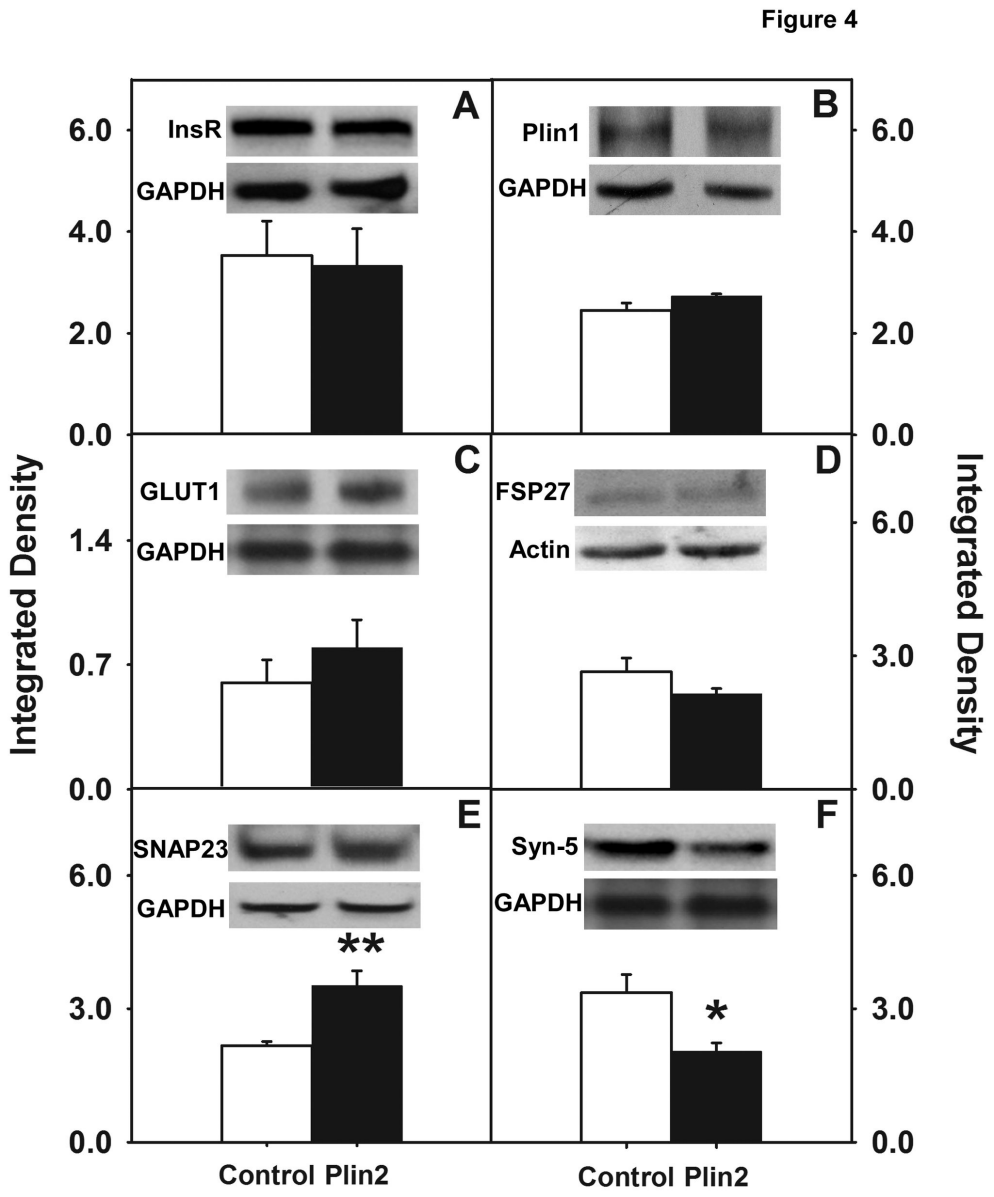
Relative expression levels of key proteins involved in lipid droplet formation, glucose uptake and transport. Cell homogenates from Plin2 overexpressing and control cells were probed with antibodies against the following proteins: insulin receptor (A), Plin1 (B), GLUT1 (C), FSP27 (D), SNAP23 (E), and syntaxin-5 (F). Expression levels were quantified as described in the Method section, normalized to the housekeeper gene GAPDH. Values represent mean ± SEM (n=3-5). (*) indicates p < 0.01 and (**) indicates p < 0.001 as compared to control. Insets: Representative Western blots showing relative protein expression of proteins of interest and housekeeping gene (GAPDH or actin).

To explore the role Plin2 may play in regulating cellular metabolism, Western blot analyses were performed using antibodies against the following proteins: insulin receptor (Figure 4A), Plin1 (Figure 4), GLUT1 (Figure 4C), FSP27 (Figure 4D), SNAP23 (Figure 4E), syntaxin-5 (Figure 4F) and VAMP4 (data not shown). Based on the results from multiple Western blots, it was demonstrated that expression levels of the insulin receptor, Plin1, GLUT1 (the primary glucose transporter in L cells), FSP27, and VAMP4 were not significantly changed in Plin2 overexpression cells when compared to control cells. In contrast, levels of SNAP23 were increased 1.6-fold (p< 0.001) while syntaxin-5 was reduced 1.7 fold (p < 0.01). Since SNAP23, along with syntaxin-5 and VAMP4, is involved in the docking and fusion of glucose transporter vehicles to plasma membrane [30] increased expression of SNAP23 would be expected to enhance glucose uptake, not inhibit as was observed in the present work. On the other hand, decreased expression of syntaxin-5 was more in line with results found in skeletal muscle where decreased syntaxin-5 was shown to impair glucose uptake [32]. Since SNAP23, syntaxin-5, and VAMP4 are present not only at the plasma membrane but also on the lipid droplet surface and are implicated in lipid droplet fusion and growth [32], it may be suggested that expression levels were not as important as the localization and partitioning of these proteins in the cell. In summary, cellular glucose uptake was significantly inhibited in cells overexpressing Plin2, effects that could not be attributed to changes in levels of the insulin receptor, GLUT1, or other lipid droplet-associated proteins including FSP27 and Plin1.

### Intracellular localization and FRET analysis of SNAP23 with Plin2 or GLUT1

**Figure 5:**
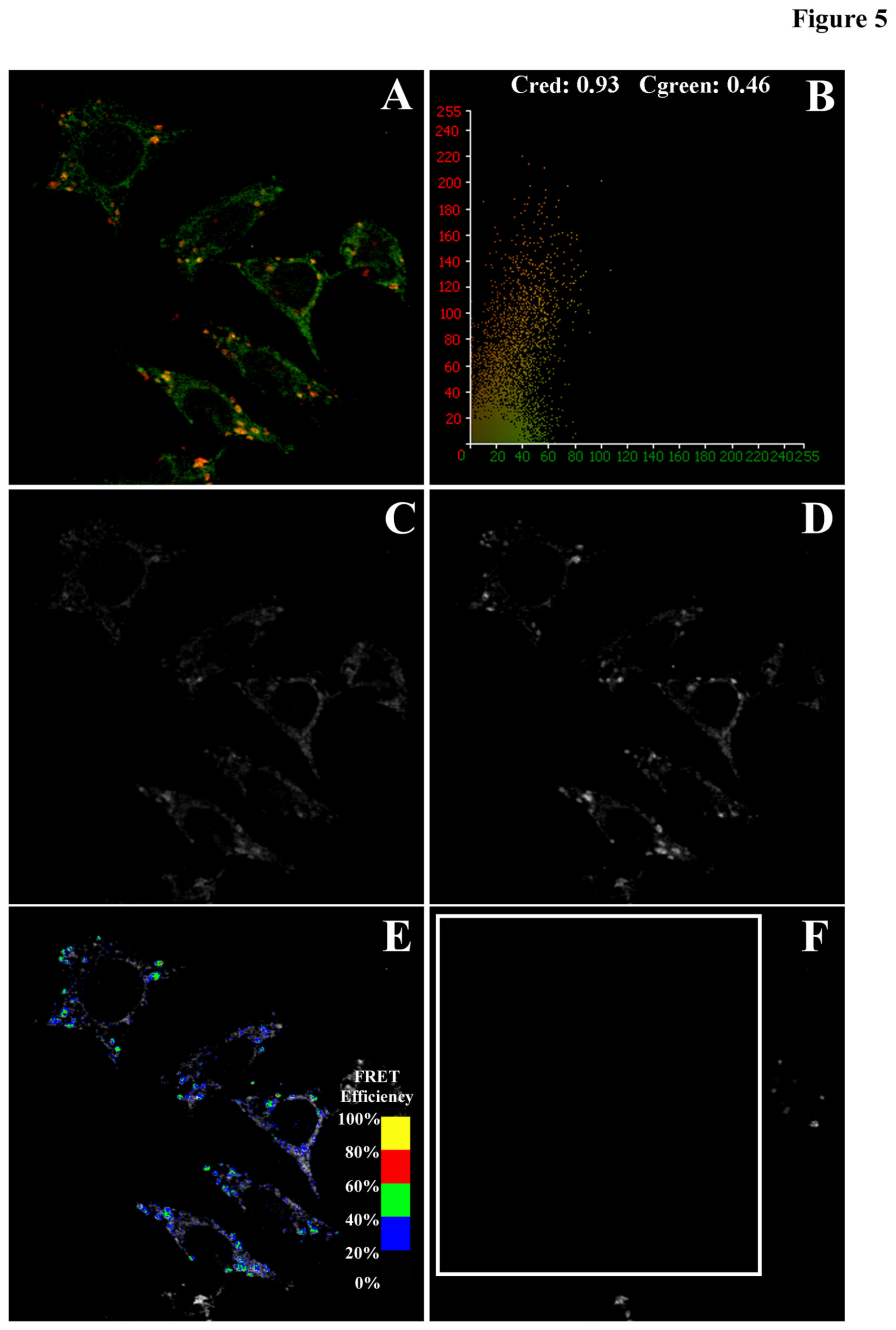
Co-localization and FRET imaging between Cy3-labeled SNAP23 and Cy5-labeled Plin2. Confocal images of Cy3-labeled SNAP23 and Cy5-labeled Plin2 were examined to determine co-localization (A) and FRET efficiencies, *E* between the fluorescently labeled proteins. The extent of co-localization was shown graphically in a pixel fluorogram (B) to reveal Cy3-SNAP23 (arbitrarily placed in the green channel) co-stained with Cy5-Plin2 (red channel) in yellow-to-orange areas where both probes co-localized. FRET efficiency maps were generated from the following images: donor emission image of Cy3-SNAP23 co-labeled with Cy5-Plin2 before acceptor (Cy5-Plin2) photobleaching (C); donor emission image of Cy3-SNAP23 co-labeled with Cy5-Plin2 after acceptor photobleaching (D); donor emission image of Cy3-SNAP23 after photobleaching overlaid with a pseudo-colored FRET image (E); and acceptor emission image of Cy5-Plin2 after photobleaching (F). Cells were imaged and FRET efficiency images generated as described in the Method section. The FRET overlay was pseudo-colored to visualize regions of higher and lower FRET as shown by the inset color scale (E).

**Figure 6:**
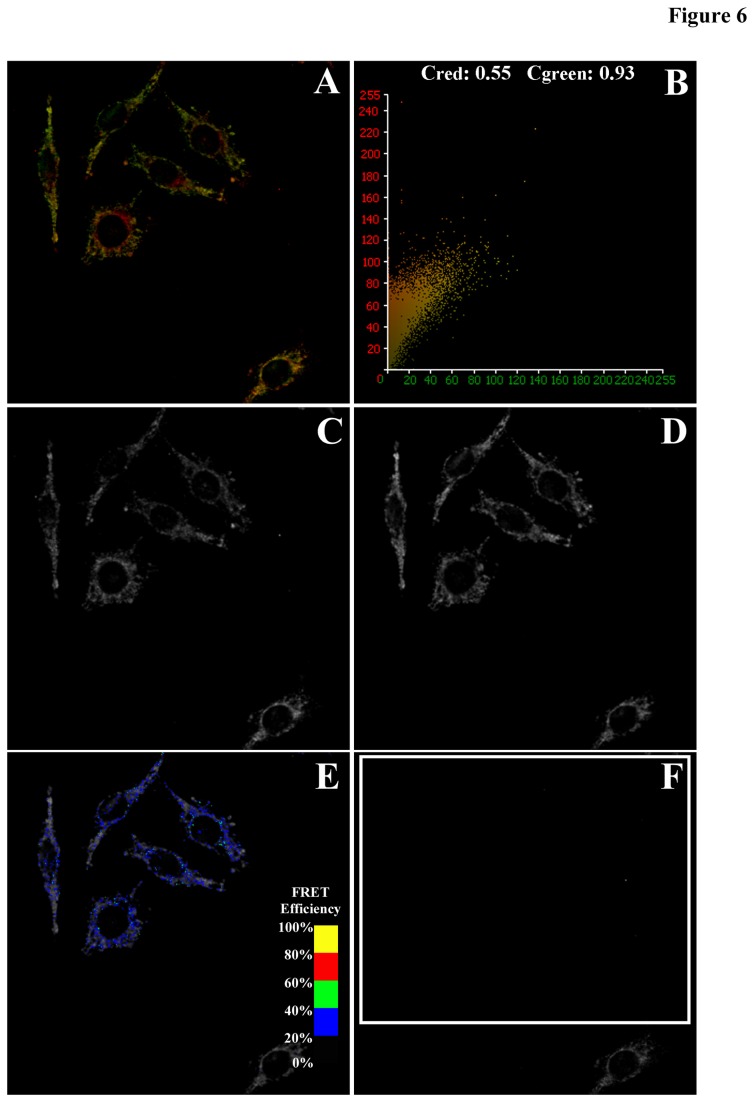
Co-localization and FRET imaging between Cy3-labeled SNAP23 and Cy5-labeled GLUT1. Confocal images of Cy3-labeled SNAP23 and Cy5-labeled GLUT1 were examined by laser scanning confocal microscopy. Co-localization of Cy3-SNAP23 (green) with Cy5-GLUT1 (red) revealed yellow-to-orange areas where both probes overlapped (A). The extent of co-localization was shown in a pixel fluorogram (B). FRET efficiency maps were generated from the following images: donor emission image of Cy3-SNAP23 co-labeled with the acceptor Cy5-GLUT1 before photobleaching (C); donor emission image of Cy3-SNAP23 co-labeled with Cy5-GLUT1 after acceptor photobleaching (D); donor emission image of Cy3-SNAP23 after photobleaching overlaid with a pseudo-colored FRET image (E); and acceptor emission image of Cy5-GLUT1 after photobleaching (F). Cells were imaged and FRET efficiency images generated as described in the Method section. The FRET overlay was pseudo-colored to visualize regions of higher and lower FRET as shown by the inset color scale (E).

The intracellular localization and targeting of SNAP23 with Plin2 and GLUT1 was examined next in a series of co-localization and FRET imaging experiments in order to determine: (i) the localization and partitioning of SNAP23 in the cell and (ii) the effect of Plin2 overexpression on SNAP23 intracellular localization. Simultaneous acquisition of confocal images of Cy3-labeled SNAP23 (green channel) with Cy5-labeled Plin2 (red channel) in control L cells revealed areas of high intensity, co-localized signals (yellow-to-orange) that were identified by morphology as lipid droplets (Figure 5A). In the representative pixel fluorogram, the correlation coefficient showed approximately 93% of the Cy5-labeled Plin2 (red) co-localized with the Cy3-labeled SNAP23 (green), while only 46% of Cy3-SNAP23 co-localized with the Cy3-labeled Plin2 (Figure 5B). As Plin2 localized primarily on the lipid droplet surface, these results indicated that SNAP23 targeted to lipid droplets as well as other intracellular locations. Similarly, control L cells were labeled with Cy3-SNAP23 (green) and Cy5-GLUT1 (red) to show co-localization in cytosolic and plasma membrane compartments of the cells as yellow to orange staining in areas of overlap (Figure 6A). From the representative pixel fluorogram (Figure 6B) approximately 55% of the Cy5-labeled GLUT1 (red) co-localized with the Cy3-labeled SNAP23 (green), while 93% of Cy3-SNAP23 (green) co-localized with Cy5-GLUT1 (red). Taken together, these results indicated that the majority of SNAP23 co-localized with GLUT1 but GLUT1 could also be found in areas without SNAP23 present.

Over all, the data pointed to a strong spatial overlap between SNAP 23 and Plin2 and also with SNAP23 and GLUT1 to within the limits of optical resolution (2,200 Å). However, co-localization studies could not provide the optical resolution to infer whether these proteins were in close enough proximity to interact and/or co-exist in a complex. Therefore, fluorescence resonance energy transfer (FRET) studies were performed as described in [13,55] to measure direct protein–protein interactions at the subcellular level to within 10-100 Å. FRET was measured as the increase in donor (Cy3-labeled SNAP23) emission upon photobleaching of the acceptor (Cy5-labeled Plin2 or GLUT1). For the Cy3-SNAP23/Cy5-Plin2 FRET experiment, emission of the Cy3-labeled SNAP23 was imaged by exciting the Cy3 label at 559 nm excitation (Figure 5C) followed by exciting the cells at 633 nm to image the emission of Cy5-labeled Plin2 (Figure 5D) with fluorescence bleed-through to other channels minimized as necessary. Cells in the desired field (indicated by the white boxed region in Figure 5F) were then photobleached by repeated scanning at 633 nm until no Cy5 signal was detected (Figure 5F). Photobleaching neither affected the photostability of the donor nor caused any non-specific increase in donor density as measured in cells labeled with the donor alone and subjected to acceptor photobleaching (data not shown). One cell was left outside the photobleached area to serve as a bleaching control. FRET was measured by comparing the Cy3 emission intensities before (Figure 5C) and after (Figure 5D) photobleaching as described in the Methods section. The mean FRET efficiency *E* and distance *R* between SNAP23 and Plin2 were calculated to equal 49 + 1% and 51.3 + 0.4 Å, respectively (Table 2) indicating the close proximity of SNAP23 to Plin2 in the cell. FRET efficiency images (Figure 5E, color overlay) were generated next in order to visualize the areas of the cell where FRET occurred. FRET efficiencies (*E*) were scaled to visualize regions of lower (black to blue) and higher (green to yellow) FRET (Figure 5E, inset color scale). With Cy3-SNAP23/Cy5-Plin2 labeled cells, areas of high intensity identified by morphology as lipid droplets showed *E* in the range of 45-50% (blue to cyan on the FRET inset color scale), consistent with the measured value of the mean *E* equal to 49%. In similar fashion, FRET was performed on cells labeled with Cy3-SNAP23/Cy5-GLUT1 (Figure 6), where the mean *E* and *R* were calculated as 27 + 1% and 60.4 + 0.3 Å, respectively (Table 2). FRET efficiency images were generated to show FRET occurring between SNAP23 and GLUT1 in more diffuse areas of the cytoplasm outside lipid droplets (Figure 6E) with *E* equal to 25-30% (blue on the FRET inset color scale). Taken together, these results indicated that SNAP23 interacted directly with Plin2 at the lipid droplet surface and GLUT1 in the cytoplasm as it partitioned between lipid droplets and the plasma membrane, but was in closer proximity with Plin2 (p<0.001, Table 2).

**Table 2:** FRET efficiency *E* and distance *R* between Cy3 labeled SNAP23 and Cy5-labeled Plin2 or GLUT1.

FRET Pair	*E* (%)	*R* (Å)
SNAP23/Plin2	49 ± 1*	51.3 ± 0.4*
SNAP23/GLUT1	27 ± 1	60.4 ± 0.3

FRET efficiency *E* and distance *R* values were determined as described in the Method section. Values are mean + SE; n = 45-60 cells. (*) indicates p < 0.001 as compared to the SNAP23/GLUT1 FRET pair.

### Effect of Plin2 overexpression on SNAP23 partitioning between lipid droplets and the plasma membrane

**Figure 7:**
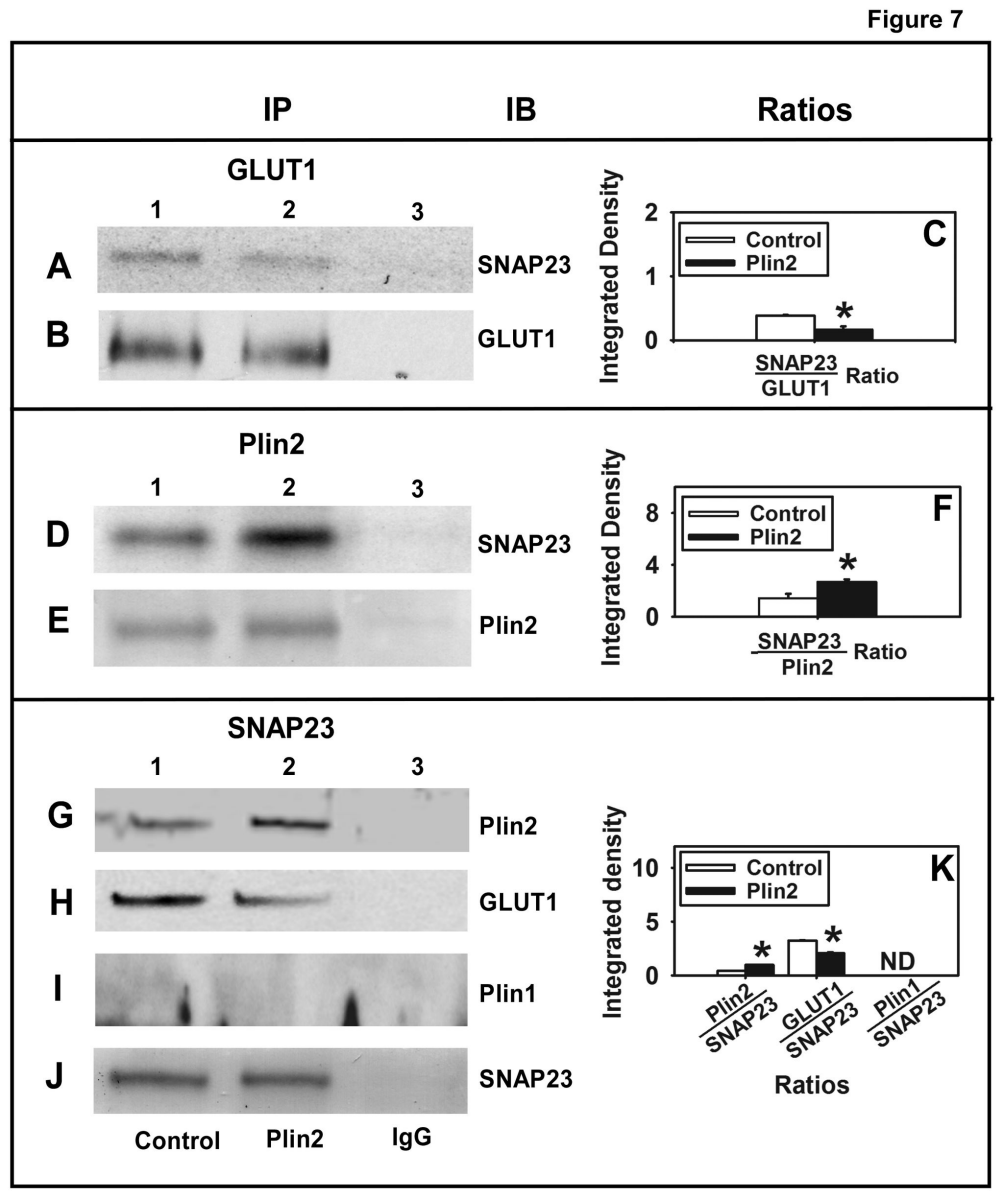
Co-immunoprecipitation of SNAP23 with GLUT1, Plin2, or Plin1. The native proteins in cell homogenates from Plin2 overexpressing and control cells were co-immunoprecipitated as described in the Methods section. To examine SNAP23/GLUT1 interactions, GLUT1 was immunoprecipitated using anti-GLUT1. Levels of SNAP23 (A) in the immunoprecipitate from control (lane 1) and PLIN2 overexpressing cells (lane 2) were analyzed by immunoblotting with anti-SNAP23. Equal immunoprecipitation of GLUT1 was verified by immunoblotting with anti-GLUT1 (B). The ratio of SNAP23 immunoprecipitated with GLUT1 was calculated from the integrated density values from Western blots (C). To analyze SNAP23/Plin2 interactions, Plin2 was immunoprecipitated with anti-Plin2 and levels of SNAP23 (D) were detected by immunoblotting with anti-SNAP23. Equal immunoprecipitation of Plin2 was verified by immunoblotting with anti-Plin2 (E). The ratio of SNAP23 immunoprecipitated with GLUT1 was calculated from the integrated density values from Western blots (F). Reverse immunoprecipitation experiments were also performed. SNAP23 was immunoprecipitated with anti-SNAP23 and levels of Plin2 (G), GLUT1 (H), and Plin1 (I) in the immunoprecipitate were analyzed by Western blotting. Equal immunoprecipitation of SNAP23 was verified by immunoblotting (J). The ratio of Plin2, GLUT1 and Plin1 immunoprecipitated with SNAP23 was calculated from the integrated density values from Western blots (K). Immunoprecipitate obtained using secondary IgG antibodies (lane 3) were used as negative controls for each set. (*) indicates p < 0.05 as compared to control.

SNARE proteins like SNAP23 (also syntaxins and VAMP4) play a critical role in moving GLUT vesicles to the plasma membrane and are also present at the surface of lipid droplets [32]. In the present work levels of SNAP23 were increased in the Plin2 overexpression cells, results counterintuitive to the observed inhibition of glucose uptake. Since the amount of SNAP23 available may be less important than the location of SNAP23 in the cell, co-localization and co-immunoprecipitation (co-IP) techniques were performed to determine the extent of SNAP23 overlap with Plin2 in lipid droplets versus GLUT1 in the cytoplasm/plasma membrane when Plin2 expression was increased. For the co-IP experiments, cells were immunoprecipitated with the GLUT1 antibody in control cells (Figure 7, lane 1) and Plin2 overexpression cells (Figure 7, lane 2) and then immunoblotted with anti-SNAP23 to show that interactions between SNAP23 and GLUT1 were decreased significantly 42% (p < 0.5) in Plin2 overexpression cells (Figure 7A; lanes 1 vs 2, Figure 7C). Cells were also immunoblotted with the GLUT1 antibody to show that levels of GLUT1 immunoprecipitated in the assay were similar (Figure 7B; lanes 1 vs 2). Plin2 was next immunoprecipitated from each cell type and immunoblotted with SNAP23 to show an 85% increase in SNAP23 levels in Plin2 overexpression cells (Figure 7D; lanes 1 vs 2, Figure 7F). These results suggested that SNAP23/Plin2 interactions were enhanced when Plin2 was overexpressed. The changes observed in the amount of SNAP23 co-precipitated were not due to differences in the amount of Plin2 immunoprecipitated since immunoblotting with Plin2 antibody following immunoprecipitation showed equal levels of Plin2 (Figure 7E, lanes 1 vs 2) indicating the amount of Plin2 immunoprecipitated was similar in both cell types. Experiments were performed in the reverse by immunoprecipitating SNAP23 and immunoblotting with each antibody to show: (i) Enhanced interaction (2 fold) between Plin2 and SNAP23 (Figure 7G, lanes 1 vs 2, Figure 7K); (ii) Reduced interaction (37%) between GLUT1 and SNAP23 (Figure 7H, lane 1 vs 2, Figure 7K); (iii) No interaction of SNAP23 with Plin1 (Figure 7I, lane 1 vs 2); and (iv) Levels of SNAP23 immunoprecipitated in the assay were similar in the Plin2 overexpression and control cells (Figure 7J, lane 1 vs 2). Taken together, these data demonstrate increased SNAP23/Plin2 association and SNAP23 partitioning to lipid droplets in Plin2 overexpression cells. Thus, despite increased SNAP23 expression in cells overexpressing Plin2, increased association and partitioning of SNAP23 with Plin2 reduced the availability of SNAP23 to interact with GLUT1 vesicles, thereby reducing 2-NBD-glucose uptake in cells with increased Plin2 expression.

**Figure 8:**
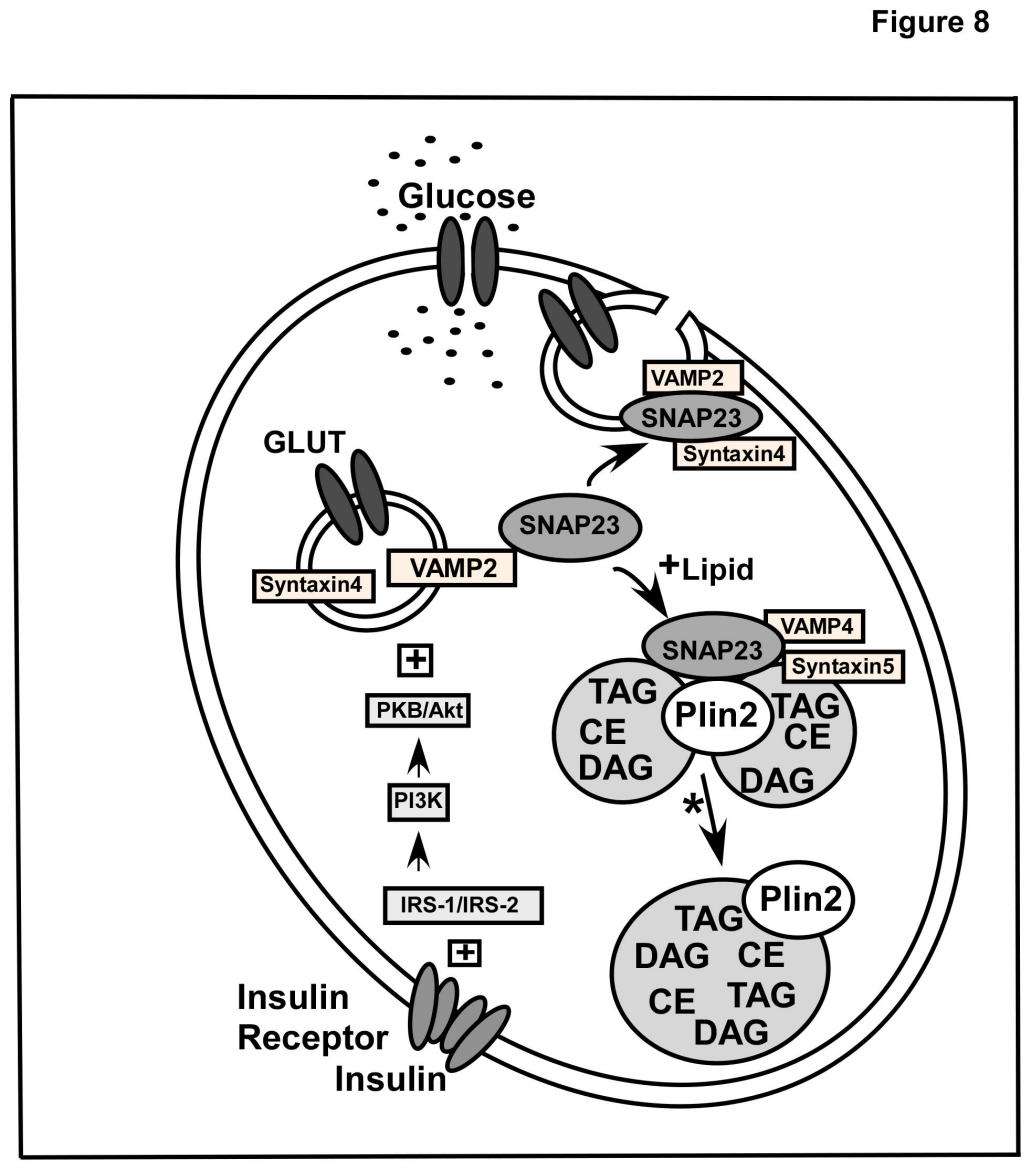
Schematic diagram for SNARE-mediated regulation of glucose transport in Plin2 overexpression cells. Key regulators involved in glucose uptake and transport are illustrated. Glucose transporters (GLUT) are involved in the entry of glucose into the cells. In the absence of insulin stimulation, these transporters predominantly reside in vesicular structures that move slowly from the cytoplasm to PM. Upon insulin stimulation GLUT-containing vesicles translocate, dock, and fuse with the plasma membrane through the action of SNARE fusion machinery proteins including SNAP23, Syntaxin-5, and VAMP4. Similar proteins (SNAP23, Syntaxin-4, and VAMP2) are also present on the lipid droplet surface along with Plin2 which directly interacts with SNAP23. Under conditions of excess lipid storage that increase lipid droplet formation and Plin2 expression, SNAP23 is retained on the lipid droplet by enhanced interaction between Plin2 and SNAP23. A decreased interaction between SNAP23 with other SNARE proteins at the plasma membrane results in decreased glucose uptake. * Studies that show SNAP23 participate in Plin2-coated lipid droplet fusion are described in [32,72].

The effect of Plin2 expression on interactions between SNAP23/Plin2 versus SNAP23/GLUT1 was also examined by calculating the percent co-localization of each image pair in control and Plin2 overexpression cells. As shown in Table 3, when Plin2 was increased, the percent co-localization of SNAP23 with Plin2 significantly increased from 30% to 41% and the amount of Plin2 interacting with SNAP23 increased from 76 to 84%. In contrast, the amount of SNAP23 co-localized with GLUT1 decreased to 26% from 56% and to 77% from 91% in the reverse experiment when Plin2 levels were increased (Table 3). These results suggested that Plin2 overexpression led to significantly increased interactions of SNAP23 with Plin2 but diminished contact with GLUT1. In all, these results indicated that when Plin2 levels were increased SNAP23 partitioned to a greater extent to lipid droplets in close association with Plin2 at the expense of SNAP23/GLUT1 interactions. Taken together, results presented herein suggest that SNAP23 is retained on the lipid droplet surface by enhanced interaction with Plin2 under conditions of lipid accumulation when Plin2 expression is increased, leading to decreased interaction at the plasma membrane and decreased glucose uptake. A schematic diagram of these results is depicted in Figure 8.

**Table 3:** Percent co-localization of SNAP23 interactions with Plin2 versus GLUT1 in control and Plin2 over expression cells.

	% Co-localization	
Colocalization Pair	Control	Plin2
*SNAP23:Plin2*		
% SNAP23 w/ Plin2	30 ± 3	41 ± 4*
%Plin2 w/ SNAP23	76 ± 2	84 ± 2*
*SNAP23:GLUT1*		
% SNAP23 w/ GLUT1	56 ± 4	26 ± 9*
% GLUT1 w/ SNAP23	91 ± 3	77 ± 5*

Values represent mean ± SEM. Percent co-localization was calculated from Olympus Fluoview software as described in the Method section. (*) indicates p < 0.05 as compared to the control cells.

## Discussion

While dysregulation of lipid metabolism in lipid droplet function has been implicated in the development of obesity and insulin resistant type 2 diabetes [56], the functional significance of lipid droplets and associated proteins such as Plin2 in the regulation of glucose uptake and metabolism remains largely unknown. This is of interest since several studies with rodent models and clinical subjects suggest that Plin2 may influence glucose metabolism. For instance, leptin-deficient Zucker diabetic fatty (ZDF) rats fed a high fat diet showed increased expression of Plin2 in muscle during the progression of diabetes [22], while Varela et al. [23] showed that development of insulin resistance was reduced by treatment with anti-sense oligonucleotides specific to Plin2. In line with this, leptin deficient obese (Lep^ob/ob^) mice crossed with mice deficient in full length Plin2 showed improved glucose tolerance and enhanced glucose disposal rate compared to their obese Lep^ob/ob^ counterparts [24,25]. Another study showed that treatment of diabetic subjects with rosiglitazone, a known insulin sensitizer reduced levels of myocellular Plin2 content [22]. Moreover, Faleck et al. [33] showed treatment of Plin2 anti-sense oligonucleotides in mice decreased palmitoic acid-induced insulin secretion from β-cells, suggesting an involvement of Plin2 in insulin secretion from pancreatic β-cells. In general, these studies suggested that Plin2 can influence cellular glucose metabolism by regulating both insulin secretion from β-cells and insulin sensitivity of peripheral cells. However, while these reports supported a link between Plin2 and insulin resistance leading to diabetes, no direct experimental evidence was given to explain these observations at the cellular level. In the current study this issue was addressed by examining the effect of Plin2 overexpression on glucose uptake and metabolism. Results presented herein show for the first time that Plin2 overexpression inhibited cellular glucose uptake 1.8-fold in stably transfected L cells. The inhibitory effect of Plin2 was further confirmed by using a radiolabeled glucose analogue [^3^H]-2-deoxyglucose, wherein a 2-fold decrease was observed. In contrast, a complementary RNAi-mediated knockdown approach showed nearly a 2-fold increase in glucose uptake in both L cells and differentiated 3T3-L1 cells with decreased Plin2 expression. Based on the kinetic parameters derived from the fitted uptake curves, Plin2 expression regulated the extent of glucose entering the cells, as reflected by the decreased Fmax and also influenced the speed of uptake, as shown by the slower initial rate of uptake in Plin2 overexpression cells. Treatment of cells with cytochalasin B, a GLUT inhibitor decreased glucose uptake while treatment with insulin increased uptake parameters. These results confirmed that 2-NBD-Glucose uptake and transport in L cells was mediated by glucose transporters and insulin. This was of some importance since translocation of GLUT containing secretory vesicle (GSV) from intracellular locations and fusion to the plasma membrane are critical steps in the regulation of glucose uptake. In the present work, a decrease in cellular glucose uptake was observed in Plin2 overexpression cells indicating a potential role for Plin2 in regulating this process.

Since glucose uptake and transport is dependent not only on the insulin receptor and GLUT proteins but also on SNARE proteins involved in the vesicular fusion machinery that facilitate glucose uptake into the cell, it was of interest to examine expression levels of not only the insulin receptor and GLUT proteins, but also SNARE proteins such SNAP23, syntaxin-5, and VAMP4 that are notably present on lipid droplets [32]. It was also important to examine levels of fat specific protein 27 (FSP27) in Plin2 overexpression cells since FSP27 can facilitate lipid droplet fusion [57–59] and since FSP27 knockout mice showed improved insulin sensitivity [60]. Moreover, FSP27 is enriched at contact sites between two lipid droplets and has been shown to facilitate the transfer of lipids on lipid droplet surface [61]. Levels of Plin1 were also examined since recent reports have implicated a potential role for Plin1 in the development of insulin resistance [62]. In one study, older Plin1 null mice were lean and resistant to diet-induced obesity yet developed insulin resistance [14,63]. In other work, Puri et al. [64] reported that Plin1 mRNA levels were decreased in visceral and subcutaneous adipose tissues of insulin resistant obese individuals. Conversely, Kern et al. [65] reported that obese patients with increased Plin1 in adipocytes interspersed in skeletal muscle did not show any change in insulin sensitivity. In addition, FSP27 has been shown to interact with Plin1 [66] while Plin1 increases the activity of FSP27 in the formation of unilocular LDs in adipocytes [67]. In the present work, analysis of multiple Western blots revealed that expression levels of the insulin receptor, GLUT1 (the primary glucose transporter in L cells), FSP27, and Plin1 were not significantly changed in Plin2 overexpression cells when compared to control cells, ruling out effects due to altered expression of these proteins. In contrast, expression of SNAP23 was increased in Plin2 overexpression cells, while levels of syntaxin-5 were decreased with no observed change in the expression of VAMP4. These results were consistent with one study that showed RNAi-mediated knock down of syntaxin-5 resulted in a defective insulin action as reflected by a decrease in insulin-dependent Akt phosphorylation in cultured human skeletal-muscle cells [68]. However, another study found increased SNAP23 expression in the skeletal muscle of type-2 diabetic patients [69]. In the present work, reduction in syntaxin-5 levels may have slowed down the process of glucose transporter vesicle fusion to the membrane, leading to the observed decreased initial rate. However, since SNAP23 and syntaxin-5 are involved in the docking and fusion of glucose transporters to the plasma membrane to allow glucose uptake [30], increased SNAP23 expression would be expected to enhance glucose uptake, contrary to the observed results. One explanation for these counterintuitive results could be that expression levels may not be as important as cellular localization and partitioning of these proteins. It was recently reported that SNAP23, syntaxin-5, and VAMP4 were present in lipid droplets and were essential for lipid droplet fusion and growth [32]. Hence it is possible that lipid droplets may be sharing and/or competing with the cell’s vesicular fusion machinery for the SNARE complex proteins. To explore this possibility, interactions between Plin2 and SNAP23 (and also SNAP23 with GLUT1) were analyzed by FRET imaging to determine if SNAP23 was in direct contact with Plin2 on the lipid droplet surface. FRET, a technique based on examining the non-radiative transmission of energy from an excited donor molecule to a nearby acceptor molecule that is dependent on the dipole-dipole interactions of fluorescent donor to acceptor molecules is capable of detecting molecule-molecule interactions at distances in the range of 10-100 Å. In the case of Cy3-labeled SNAP23 and Cy5-labeld Plin2, the measured intermolecular distance *R* between the two molecules was 51.3 + 0.4 Å (Table 3), indicating the two proteins were in close physical association on the lipid droplet surface. Distances between the Cy3/Cy5-labeled SNAP23/GLUT1 pair were 60.4 + 0.3 Å, indicating close intermolecular distances as well, but significantly more than the distance between SNAP23/Plin2 (n=45-60 cells, P<0.001). Measured *E* values, representing the efficiency of energy transfer between donor and acceptor and dependent on the distance *R* and the orientation of the donor and acceptor molecules was also determined. The mean *E* was 49 ± 1% for the SNAP23/Plin2 FRET pair and 27±1% for SNAP23/GLUT1, indicating significantly lower energy transfer occurred between the SNAP23/GLUT1 pair (n=45-60 cells, P<0.001). *E* was also quantified on a pixel-by-pixel basis in FRET image maps to identify specific areas within the cell where the percentage of *E* was highest. For the SNAP23/Plin2 pair, areas of the cell overlapping with lipid droplets showed the highest *E* values ranging from 40–50% on the FRET efficiency scale (Figure 5E). With the SNAP23/GLUT1 pair, FRET occurred in diffused areas throughout the cell with *E* significantly lower (in the 20-30% range) than observed with SNAP23/Plin2 pair (Figure 6E). Thus, FRET imaging studies indicated a direct interaction between SNAP23 and Plin2 on the surface of lipid droplets and a more diffuse pattern of SNAP23/GLUT1 interaction in areas outside the lipid droplet.

The ability of SNAP23 to partition selectively between lipid droplets and the plasma membrane was also examined in a series of co-IP and co-localization experiments in order to explain how enhanced expression of Plin2 and SNAP23 could lead to decreased cellular glucose uptake in transfected cells. Based on the results from multiple co-IP experiments, interactions between Plin2 and SNAP23 were enhanced when expression of Plin2 was increased. Concomitantly, partitioning of SNAP23 with GLUT1 was decreased in Plin2 overexpression cells, suggesting that in the presence of Plin2 overexpression, SNAP23 was less available for vesicular fusion at the plasma membrane resulting in less cellular glucose uptake. In keeping with these results, when the percent co-localization and extent of overlap between SNAP23/Plin2 versus SNAP23/GLUT1 were examined, the percent co-localization and overlap coefficient between the SNAP23/Plin2 pair were significantly increased while SNAP23/GLUT1 interactions were significantly decreased when Plin2 was present at higher levels. Taken together, these results suggest that when Plin2 levels were increased SNAP23 partitioned to a greater extent to Plin2-associated lipid droplets at the expense of SNAP23/GLUT1 interactions. It should be noted that the current work did not show direct evidence that increased SNAP23/Plin2 interactions correlated with decreased GLUT1 on the plasma membrane and this represents a limitation to the study. Moreover, mechanisms underlying the relationship between insulin dependent GLUT4 and Plin2-coated LD could not be addressed based on the data presented. Future studies are planned to investigate these issues in more detail. Nevertheless, other work has shown that treatment of cardiac cells with oleic acid (which increased lipid droplet size and number) significantly reduced SNAP23 at the plasma membrane while increasing its presence in lipid droplets, resulting in reduced insulin-stimulated glucose uptake [32]. Moreover, increased partitioning of SNAP23 to cytosolic/microsomal compartments and reduced SNAP23 expression at the plasma membrane was also observed in studies using muscle cells of type-2 diabetic patients [69]. In keeping with this, it was recently demonstrated that α-synuclein null mice that are resistance to diet induced obesity showed decreased lipid droplet size because of reduced SNARE complex formation in adipocytes, further highlighting the role of SNARE proteins in lipid droplet formation and fusion [70]. It should be noted however, another report revealed FSP27 and Plin1 interact with each other and are involved in the formation of unilocular lipid droplets [66]. Interestingly, while FSP27 interacts with Plin1, it does not seem to interact with Plin2 as studied by co-immunoprecipitation experiments [67]. Hence, given the mutually exclusive presence of Plin1 and Plin2 on lipid droplets, it is possible that FSP27/Plin1 interactions may be primarily involved in the formation of unilocular lipid droplets, while SNAP23/Plin2 interactions may direct the formation of multilocular lipid droplets. Further studies are warranted to test if such distinct mechanisms exist in lipid droplet fusion. In this regard, it is interesting to note that knock down of SNAP23 in S2 cells had no effect on unilocular LD formation [71]. Although further studies may be needed to ascertain the role of SNAP23 in lipid droplet fusion, data from the present work suggests that Plin2 influences cellular glucose uptake and transport by re-directing SNAP23 to lipid droplets.

In summary, the current investigation provides a fresh mechanistic view of the role of lipid droplets and associated proteins in cellular metabolism by demonstrating the following novel insights. First, insulin-mediated cellular glucose uptake inversely correlates to Plin2 expression in cells without altered levels of the insulin receptor, glucose transporters, or other lipid droplet-associated proteins. Second, levels of the SNARE complex protein SNAP23 was significantly increased in Plin2 overexpression cells, results at odds with the decreased uptake data but explained by increased partitioning of SNAP23 with Plin2 on the lipid droplet surface where SNAP23 was shown to be in direct physical association with Plin2. Concomitantly, interactions with SNAP23 and GLUT1 were decreased in Plin2 overexpression cells, suggesting that expression levels of Plin2 may regulate the availability of SNAP23 for vesicular fusion at the plasma membrane. Taken together, these data suggest that Plin2 influences cellular glucose uptake and transport by interacting with, and regulating cellular targeting of various SNARE proteins. Since insulin resistance can be characterized by inefficient glucose uptake into muscle and fat cells, the observed changes in insulin-stimulated glucose uptake due to altered Plin2 expression gives support to the premise that Plin2 may play an important physiological role in regulating cellular insulin resistance.

## References

[1] Samuel VT, Petersen KF, Shulman GI (2010) Lipid-induced insulin resistance: unravelling the mechanism. Lancet 375: 2267-2277. doi:10.1016/S0140-6736(10)60408-4. PubMed: 20609972. 20609972 10.1016/S0140-6736(10)60408-4 PMC2995547

[2] Fujimoto T, Parton RG (2011) Not just fat: The structure and function of the lipid droplet. Cold Spring Harb Perspect Biol 3: a004838. doi:10.1101/cshperspect.a004838. PubMed: 21421923. 21421923 10.1101/cshperspect.a004838 PMC3039932

[3] Bickel PE, Tansey JT, Welte MA (2009) PAT proteins, an ancient family of lipid droplet proteins that regulate cellular lipid stores. Biochim Biophys Acta 1791: 419-440. doi:10.1016/j.bbalip.2009.04.002. PubMed: 19375517. 19375517 10.1016/j.bbalip.2009.04.002 PMC2782626

[4] Ahmadian M, Duncan RE, Sul HS (2009) The skinny on fat: lipolysis and fatty acid utilization in adipocytes. Trends Endocrinol Metab 20: 424-428. doi:10.1016/j.tem.2009.06.002. PubMed: 19796963. 19796963 10.1016/j.tem.2009.06.002 PMC2764815

[5] Zechner R, Zimmermann R, Eichmann TO, Kohlwein SD, Haemmerle G et al. (2012) Fat Signals- Lipases and lipolysis in lipid metabolism and signaling. Cell Metab 15: 279-291. doi:10.1016/j.cmet.2011.12.018. PubMed: 22405066. 22405066 10.1016/j.cmet.2011.12.018 PMC3314979

[6] Wolins NE, Quaynor BK, Skinner JR, Schoenfish MJ, Tzekov A et al. (2005) S3-12, adipophilin and TIP47 package lipid in adipocytes. J Biol Chem 280: 19146-19155. doi:10.1074/jbc.M500978200. PubMed: 15731108. 15731108 10.1074/jbc.M500978200

[7] Londos C, Brasaemle DL, Schultz CJ, Segrest JP, Kimmel AR (1999) Perilipins, ADRP, and other proteins that associate with intracellular neutral lipid droplets in animal cells. Cell Dev Biol 10: 51-58. doi:10.1006/scdb.1998.0275. PubMed: 10355028. 10.1006/scdb.1998.0275 10355028

[8] Wolins NE, Quaynor BK, Skinner JR, Tzekov A, Croce MA et al. (2006) OXPAT/PAT-1 is a PPAR-induced lipid droplet protein that promotes fatty acid utilization. Diabetes 55: 3418-3428. doi:10.2337/db06-0399. PubMed: 17130488. 17130488 10.2337/db06-0399

[9] Brasaemle DL, Barber T, Wolins NE, Serrero G, Blanchette-Mackie EJ et al. (1997) Adipose differentiation-related protein is an ubiquitously expressed lipid storage droplet-associated protein. J Lipid Res 38: 2249-2263. PubMed: 9392423. 9392423

[10] Larigauderie G, Cuaz-Pérolin C, Younes AB, Furman C, Lasselin C et al. (2006) Adipophilin increases triglyceride storage in human macrophages by simulation of biosynthesis and inhibition of beta-oxidation. FEBS J 273: 3498-3510. doi:10.1111/j.1742-4658.2006.05357.x. PubMed: 16884492. 16884492 10.1111/j.1742-4658.2006.05357.x

[11] Fukushima M, Enjoji M, Kohjima M, Sugimoto R, Ohta S et al. (2005) Adipose differentiation related protein induces lipid accumulation and lipid droplet formation in hepatic stellate cells. In Vitro Cell Dev Biol Anim 41: 321-324. doi:10.1290/0410069.1. PubMed: 16448220. 16448220 10.1007/s11626-005-0002-6

[12] Listenberger LL, Ostermeyer-Fay AG, Goldberg EB, Brown WJ, Brown DA (2007) Adipocyte differentiation-related protein reduces the lipid droplet association of adipose triglyceride lipase and slows triacyglycerol turnover. J Lipid Res 48: 2751-2761. doi:10.1194/jlr.M700359-JLR200. PubMed: 17872589. 17872589 10.1194/jlr.M700359-JLR200

[13] McIntosh AL, Senthivinayagam S, Moon KC, Gupta S, Lwande JS et al. (2012) Direct interaction of ADRP with lipids on the surface of lipid droplets: A live cell FRET analysis. Am J Physiol Cell Physiol 303: C728-C742. doi:10.1152/ajpcell.00448.2011. PubMed: 22744009. 22744009 10.1152/ajpcell.00448.2011 PMC3469596

[14] Tansey JT, Sztalryd C, Gruia-Gray J, Roush DL, Zee JV et al. (2002) Perilipin ablation results in a lean mouse with aberrant adipocyte lipolysis, enhanced leptin production, and resistance to diet-induced obesity. Proc Natl Acad Sci U S A 98: 6494-6499. 10.1073/pnas.101042998 PMC33496 11371650

[15] Atshaves BP, Storey SM, McIntosh AL, Petrescu AD, Lyuksyutova OI et al. (2001) Sterol carrier protein 2 expression modulates protein and lipid composition of lipid droplets. J Biol Chem 276: 25324-25335. doi:10.1074/jbc.M100560200. PubMed: 11333258. 11333258 10.1074/jbc.M100560200

[16] Atshaves BP, Starodub O, McIntosh AL, Roths JB, Kier AB et al. (2000) Sterol carrier protein-2 alters HDL-mediated cholesterol efflux. J Biol Chem 275: 36861. 10.1074/jbc.M003434200 10954705

[17] Frolov A, Petrescu A, Atshaves BP, So PTC, Gratton E et al. (2000) High density lipoprotein mediated cholesterol uptake and targeting to lipid droplets in intact L-cell fibroblasts. J Biol Chem 275: 12780. 10.1074/jbc.275.17.12769 10777574

[18] Serrero G, Frolov A, Schroeder F, Tanaka K, Gelhaar L (2000) Adipose differentiation related protein. Expression, purification of recombinant protein in E. coli and characterization of its fatty acid binding properties. Biochim Biophys Acta 1488: 245-254. doi:10.1016/S1388-1981(00)00128-1. PubMed: 11082534. 11082534 10.1016/s1388-1981(00)00128-1

[19] McIntosh AL, Storey SM, Atshaves BP (2010) Intracellular lipid droplets contain dynamic pools of sphingomyelin: ADRP binds phopholipids with high affinity. Lipids 45: 465-477. doi:10.1007/s11745-010-3424-1. PubMed: 20473576. 20473576 10.1007/s11745-010-3424-1 PMC3065392

[20] Chang BH, Li L, Paul A, Taniguchi S, Nannegari V et al. (2006) Protection against fatty liver but normal adipogenesis in mice lacking adipose differentiation-related protein. Mol Cell Biol 26: 1063-1076. doi:10.1128/MCB.26.3.1063-1076.2006. PubMed: 16428458. 16428458 10.1128/MCB.26.3.1063-1076.2006 PMC1347045

[21] McManaman JL, Bales ES, Orlicky DJ, Jackson MB, MacLean PS et al. (2013) Perilipin-2 null mice are protected against diet-induced obesity, adipose inflammation and fatty liver disease. J Lipid Res (In press). 10.1194/jlr.M035063 PMC3622329 23402988

[22] Minnaard R, Schrauwen P, Schaart G, Jorgensen JA, Lenaers E et al. (2009) Adipocyte differentiation-related protein and OXPAT in rat and human skeletal muscle: Involvement in lipid accumulation and type 2 diabetes mellitus. J Clin Endocrinol Metab 94: 4077-4085. doi:10.1210/jc.2009-0352. PubMed: 19602560. 19602560 10.1210/jc.2009-0352

[23] Varela GM, Antwi DA, Dhir R, Yin X, Singhal NS et al. (2008) Inhibition of ADRP prevents diet-induced insulin resistance. Am J Physiol Gastrointest Liver Physiol 295: G621-G628. doi:10.1152/ajpgi.90204.2008. PubMed: 18669627. 18669627 10.1152/ajpgi.90204.2008 PMC2536783

[24] Chang BH, Li L, Saha P, Chan L (2010) Absence of adipose differentiation related protein upregulates hepatic VLDL secretion, relieves hepatosteatosis, and improves whole body insulin resistance in leptin-deficient mice. J Lipid Res 51: 2132-2142. doi:10.1194/jlr.M004515. PubMed: 20424269. 20424269 10.1194/jlr.M004515 PMC2903828

[25] Russell TD, Palmer CA, Orlicky DJ, Bales ES, Chang B-J et al. (2008) Mammary glands of adipophilin-null mice produce an animo-terminally truncated form of adipophilin that mediates milk lipid droplet formation and secretion. J Lipid Res 49: 206-216. doi:10.1194/jlr.M700396-JLR200. PubMed: 17921437. 17921437 10.1194/jlr.M700396-JLR200

[26] Scheeper A, Joost HG, Schürmann A (2004) The glucose transporter families SGLT and GLUT: Molecular basis of normal and aberrant function. J Parent Ent Nutr 28: 365-372 10.1177/0148607104028005364 15449578

[27] Thorens B, Mueckler M (2010) Glucose transporters in the 21st Century. Am J Physiol Endocrinol Metab 298: E141-E145. doi:10.1152/ajpendo.00712.2009. PubMed: 20009031. 20009031 10.1152/ajpendo.00712.2009 PMC2822486

[28] Baldwin SA, Barros LF, Griffiths M (1995) Trafficking of glucose transporters-Signals and Mechanisms. Biosci Rep 15: 419-426. doi:10.1007/BF01204346. PubMed: 9156573. 9156573 10.1007/BF01204346

[29] McGowan KM, Long SD, Pekala PH (1995) Glucose transporter gene expression: regulation of transcription and mRNA stability. Pharm Therapy 66: 465-505. doi:10.1016/0163-7258(95)00007-4. PubMed: 7494856. 10.1016/0163-7258(95)00007-4 7494856

[30] Bryant NJ, Govers R, James DE (2002) Regulated transport of the glucose transporter GLUT4. Nat Rev Mol Cell Biol 3: 267-277. doi:10.1038/nrm782. PubMed: 11994746. 11994746 10.1038/nrm782

[31] Bryant NJ, Gould GW (2011) SNARE proteins underpin insulin-regulated GLUT4 traffic. Traffic 12: 657-664. doi:10.1111/j.1600-0854.2011.01163.x. PubMed: 21226814. 21226814 10.1111/j.1600-0854.2011.01163.x

[32] Boström P, Andersson L, Rutberg M, Perman J, Lidberg U et al. (2007) SNARE proteins mediate fusion between cytosolic lipid droplets and are implicated in insulin sensitivity. Nat Cell Biol 9: 1286-1293. doi:10.1038/ncb1648. PubMed: 17922004. 17922004 10.1038/ncb1648

[33] Faleck DM, Ali K, Roat R, Graham MJ, Crooke RM et al. (2010) Adipose differentiation-related protein regulates lipids and insulin in pancreatic islets. Am J Physiol Endocrinol Metab 299: E249-E257. PubMed: 20484013. 20484013 10.1152/ajpendo.00646.2009 PMC2928510

[34] Imamura M, Inoguchi T, Ikuyama S, Taniguchi S, Kobayashi K et al. (2002) ADRP stimulates lipid accumulation and lipid droplet formation in murine fibroblasts. Am J Physiol Endocrinol Metab 283: E775-E783. PubMed: 12217895. 12217895 10.1152/ajpendo.00040.2002

[35] Atshaves BP, Petrescu AD, Starodub O, Roths JB, Kier AB et al. (1999) Expression and intracellular processing of the 58 KDa SCP-x/3-oxoacyl-CoA thiolase in transfected mouse L cells. J Lipid Res 40: 610-622. PubMed: 10191285. 10191285

[36] Atshaves BP, Jefferson JR, McIntosh AL, Gallegos AM, McCann BM et al. (2007) Effect of sterol carrier protein-2 expression on sphingolipid distribution in plasma membrane lipid rafts/caveolae. Lipids 42: 871-884. doi:10.1007/s11745-007-3091-z. PubMed: 17680294. 17680294 10.1007/s11745-007-3091-z

[37] Atshaves BP, McIntosh AL, Lyuksyutova OI, Zipfel W, Webb WW et al. (2004) Liver Fatty Acid Binding Protein Gene Ablation Inhibits Branched-Chain Fatty Acid Metabolism in Cultured Primary Hepatocytes. J Biol Chem 279: 30954-30965. doi:10.1074/jbc.M313571200. PubMed: 15155724. 15155724 10.1074/jbc.M313571200

[38] Marzo A, Ghirardi P, Sardini D, Meroni G (1971) Simplified measurement of monoglycerides, diglycerides, triglycerides, and free fatty acids in biological samples. Clin Chem 17: 145-147. PubMed: 5543185. 5543185

[39] Bradford MM (1976) A rapid and sensitive method for the quantitation of microgram quantities of protein utilizing the principle of protein dye binding. Anal Biochem 72: 248-254. doi:10.1016/0003-2697(76)90527-3. PubMed: 942051. 942051 10.1016/0003-2697(76)90527-3

[40] Yamada K, Nakata M, Horimoto N, Saito M, Matsuoka H et al. (2000) Measurement of glucose uptake and intracellular calcium concentration in single, living pancreatic β-cells. J Biol Chem 275: 22278-22283. doi:10.1074/jbc.M908048199. PubMed: 10748091. 10748091 10.1074/jbc.M908048199

[41] Loaiza A, Porras OH, Barros LF (2003) Glutamate triggers rapid glucose transport stimulation in astrocytes as evidenced by real time confocal microscopy. J Neurosci 23: 7337-7342. PubMed: 12917367. 12917367 10.1523/JNEUROSCI.23-19-07337.2003 PMC6740433

[42] Nedachi T, Kanzaki M (2006) Regulation of glucose transporters by insulin and extracellular glucose in C2C12 myotubes. Am J Physiol Endocrinol Metab 29: E817-E828. PubMed: 16735448. 10.1152/ajpendo.00194.2006 16735448

[43] Ebstensen RD, Plagemann PGW (1972) Cytochalasin B: Inhibition of glucose and glucosamine transport. Proc Natl Acad Sci U S A 69: 1430-1434. doi:10.1073/pnas.69.6.1430. PubMed: 4338593. 4338593 10.1073/pnas.69.6.1430 PMC426719

[44] Atshaves BP, McIntosh AL, Martin GG, Landrock D, Payne HR et al. (2009) Overexpression of sterol carrier protein-2 differentially alters hepatic cholesterol accumulation in cholesterol-fed mice. J Lipid Res 50: 1429-1447. doi:10.1194/jlr.M900020-JLR200. PubMed: 19289417. 19289417 10.1194/jlr.M900020-JLR200 PMC2694341

[45] Willenborg M, Schmidt CK, Braun P, Landgrebe J, von Figura K et al. (2005) Mannose 6-phosphate receptors, Niemann-Pick C2 protein, and lysosomal cholesterol accumulation. J Lipid Res 46: 2559-2569. doi:10.1194/jlr.M500131-JLR200. PubMed: 16177447. 16177447 10.1194/jlr.M500131-JLR200

[46] Lu R, Wang H, Liang Z, Ku L, O’Donnell WT et al. (2004) The Fragile X Protein Control Microtubule-Associated Protein 1B Translation and Microtubule Stability in Brain Neuron Development. Proc Natl Acad Sci U S A 101: 15201-15206. doi:10.1073/pnas.0404995101. PubMed: 15475576. 15475576 10.1073/pnas.0404995101 PMC524058

[47] Bell M, Wang H, Chen H, McLenithan JC, Gong DW et al. (2008) Consequences of lipid droplet coat protein downregulation in liver cells: abnormal lipid droplet metabolism and induction of insulin resistance. Diabetes 57: 2037-2045. doi:10.2337/db07-1383. PubMed: 18487449. 18487449 10.2337/db07-1383 PMC2494696

[48] Moulder JW (1970) Glucose metabolism of L cells before and after infection with *Chlamydia psittaci* . J Bacteriol 104: 1189-1196. PubMed: 16559092. 16559092 10.1128/jb.104.3.1189-1196.1970 PMC248276

[49] Murphy EJ, Schroeder F (1997) Sterol carrier protein-2 mediated cholesterol esterification in transfected L cell fibroblasts. Biochim Biophys Acta 1345: 283-292. doi:10.1016/S0005-2760(97)00003-9. PubMed: 9150248. 9150248 10.1016/s0005-2760(97)00003-9

[50] Atshaves BP, Storey SM, Petrescu AD, Greenberg CC, Lyuksyutova OI et al. (2002) Expression of Fatty Acid Binding Proteins Inhibits Lipid Accumulation and Alters Toxicity in L-cell Fibroblasts. Am J Physiol Cell Physiol 283: C688-C703. doi:10.1152/ajpcell.00586.2001. PubMed: 12176726. 12176726 10.1152/ajpcell.00586.2001

[51] McArthur MJ, Atshaves BP, Frolov A, Foxworth WD, Kier AB et al. (1999) Cellular uptake and intracellular trafficking of long chain fatty acids. J LipRes 40: 1371-1383. PubMed: 10428973. 10428973

[52] Murphy EJ (1998) Sterol carrier protein-2 expression increases fatty acid uptake and cytoplasmic diffusion in L-cell fibroblasts. Amer J Physiol Gastrointest 275: G237-G243. 10.1152/ajpgi.1998.275.2.G237 9688650

[53] Murphy EJ, Stiles T, Schroeder F (2000) Sterol carrier protein-2 expression alters phospholipid content and fatty acyl composition in L-cell fibroblasts. J Lipid Res 41: 788-796. PubMed: 10787439. 10787439

[54] Murphy EJ, Prows DR, Jefferson JR, Incerpi S, Hertelendy ZI et al. (1996) Effect of insulin on fatty acid uptake and esterification in L-cell fibroblasts. Arch Biochem Biophys 335: 267-272. doi:10.1006/abbi.1996.0507. PubMed: 8914923. 8914923 10.1006/abbi.1996.0507

[55] Petrescu AD, Payne HR, Boedecker A, Chao H, Hertz R et al. (2003) Physical and functional interaction of acyl-CoA-binding protein with hepatocyte nuclear factor-4alpha. J Biol Chem 278: 51813-51824. doi:10.1074/jbc.M303858200. PubMed: 14530276. 14530276 10.1074/jbc.M303858200

[56] Greenberg AS, Coleman RA, Kraemer FB, McManaman JL, Obin MS et al. (2011) The role of lipid droplets in metabolic disease in rodents and humans. J Clin Invest 121: 2102-2110. doi:10.1172/JCI46069. PubMed: 21633178. 21633178 10.1172/JCI46069 PMC3104768

[57] Nishino N, Tamori Y, Tateya S, Kawaguchi T, Shibakusa T et al. (2008) FSP27 contributes to efficient energy storage in murine white adipocytes by promoting the formation of unilocular lipid droplets. J Clin Invest 118: 2808-2821. PubMed: 18654663. 18654663 10.1172/JCI34090 PMC2483680

[58] Jambunathan S, Yin J, Khan W, Tamori Y, Puri V (2011) FSP27 promotes lipid droplet clustering and then fusion to regulate triglyceride accumulation. PLOS ONE 6: e28614. doi:10.1371/journal.pone.0028614. PubMed: 22194867. 22194867 10.1371/journal.pone.0028614 PMC3237475

[59] Brasaemle DL, Ne W (2012) Packaging of fat: An evolving model of lipid droplet assembly and expansion. J Biol Chem 287: 2273-2279. doi:10.1074/jbc.R111.309088. PubMed: 22090029. 22090029 10.1074/jbc.R111.309088 PMC3268387

[60] Toh SY, Gong J, Du G, Li JZ, Yang S et al. (2008) Up-regulation of mitochondrial activity and acquirement of brown adipose tissue-like property in the white adipose tissue of FSP27 deficient mice. PLOS ONE 3: e2890. doi:10.1371/journal.pone.0002890. PubMed: 18682832. 18682832 10.1371/journal.pone.0002890 PMC2483355

[61] Gong J, Sun Z, Wu L, Xu W, Schieber N et al. (2011) FSP27 promotes lipid droplet growth by lipid exchange and transfer at lipid droplet contact sites. J Cell Biol 195: 953-963. doi:10.1083/jcb.201104142. PubMed: 22144693. 22144693 10.1083/jcb.201104142 PMC3241734

[62] Tai ES, Ordovas JM (2011) The role of perilipin in human obesity and insulin resistance Curr Opin Lipidol 18: 152-156. PubMed: 17353663. 10.1097/MOL.0b013e328086aeab 17353663

[63] Martinez-Botas J, Anderson JB, Tessier D, Lapillonne A, Chang BH et al. (2000) Absence of perilipin results in leanness and reverses obesity in Lepr dbdb mice. Nat Genet 26: 474-479. doi:10.1038/82630. PubMed: 11101849. 11101849 10.1038/82630

[64] Puri V, Ranjit S, Konda S, Nicoloro SMC, Straubhaar J et al. (2011) Cidea is associated with lipid droplets and insulin sensitivity in humans. Proc Natl Acad Sci U S A 105: 7833-7838. PubMed: 18509062. 10.1073/pnas.0802063105 PMC2409392 18509062

[65] Kern PA, Gregorio GI, Lu T, Rassouli N, Ranganathan G (2004) Perilipin expression in human adipose tissue is elevated with obesity. J Clin Endocrinol Metab 89: 1352-1358. doi:10.1210/jc.2003-031388. PubMed: 15001633. 15001633 10.1210/jc.2003-031388

[66] Grahn TH, Zhang Y, Lee MJ, Sommer AG, Mostoslavsky G et al. (2013) FSP27 and Plin2 interaction promotes the formation of large lipid droplets in human adipocytes. Biochem Biophys Res Commun 432: 296-301. doi:10.1016/j.bbrc.2013.01.113. PubMed: 23399566. 23399566 10.1016/j.bbrc.2013.01.113 PMC3595328

[67] Sun Z, Gong J, Wu H, Xu W, Wu L et al. (2013) Perilipin 1 promotes unilocular lipid droplet formation through the activation of FSP27 in adipocytes. Nat Commun 4: 1594. doi:10.1038/ncomms2581. PubMed: 23481402. 23481402 10.1038/ncomms2581 PMC3615468

[68] Boström P, Andersson L, Perkins R, Hojlund K, Borén J et al. (2009) The assembly of lipid droplets and its relation to cellular insulin sensitivity. Biochem Soc Trans 37: 981-985. doi:10.1042/BST0370981. PubMed: 19754436. 19754436 10.1042/BST0370981

[69] Boström P, Andersson L, Vind B, Håversen L, Rutberg M et al. (2010) The SNARE protein SNAP23 and the SNARE-interacting protein Mucn18c in human skeletal muscle are implicated in inuslin resistance/type 2 diabetes. Diabetes 59: 1870-1878. doi:10.2337/db09-1503. PubMed: 20460426. 20460426 10.2337/db09-1503 PMC2911056

[70] Millership S, Ninkina N, Guschina IA, Norton J, Brambilla R et al. (2012) Increased lipolysis and altered lipid homeostasis protect gamma synuclein-null mice from diet-induced obesity. Proc Natl Acad Sci U S A 109: 20943-20948. doi:10.1073/pnas.1210022110. PubMed: 23213245. 23213245 10.1073/pnas.1210022110 PMC3529034

[71] Krahmer N, Guo Y, Wilfling F, Hilger M, Lingrell S et al. (2011) Phosphatidylcholine synthesis for lipid droplet expansion is mediated by localized activation of CTP: Phosphocholine cytidylyltransferease. Cell Metab 14: 504-515. doi:10.1016/j.cmet.2011.07.013. PubMed: 21982710. 21982710 10.1016/j.cmet.2011.07.013 PMC3735358

[72] Jägerström S, Polesie S, Wickström Y, Johansson BR, Schröder HD et al. (2009) Lipid droplets interact with mitochondria using SNAP23. Cell Biol Int 33: 934-940. doi:10.1016/j.cellbi.2009.06.011. PubMed: 19524684. 19524684 10.1016/j.cellbi.2009.06.011

